# Unraveling Polysulfide's Adsorption and Electrocatalytic Conversion on Metal Oxides for Li‐S Batteries

**DOI:** 10.1002/advs.202204930

**Published:** 2022-12-11

**Authors:** Shungui Deng, Tiezhu Guo, Jakob Heier, Chuanfang (John) Zhang

**Affiliations:** ^1^ College of Materials Science & Engineering Sichuan University Chengdu 610065 China; ^2^ Laboratory for Functional Polymers Empa Swiss Federal Laboratories for Materials Science and Technology Überlandstrasse 129 Dübendorf CH‐8600 Switzerland; ^3^ Institute of Materials Science and Engineering Ecole Polytechnique Federale de Lausanne (EPFL) Station 12 Lausanne CH‐1015 Switzerland; ^4^ Key Laboratory of Multifunctional Materials and Structures Ministry of Education School of Electronic Science and Engineering Xi'an Jiaotong University Xi'an Shaanxi 710049 China

**Keywords:** catalytic conversion, heterostructure, lithium‐sulfur batteries, metal oxides, polysulfide shuttle

## Abstract

Lithium sulfur (Li—S) batteries possess high theoretical capacity and energy density, holding great promise for next generation electronics and electrical vehicles. However, the Li—S batteries development is hindered by the shuttle effect and sluggish conversion kinetics of lithium polysulfides (LiPSs). Designing highly polar materials such as metal oxides (MOs) with moderate adsorption and effective catalytic activity is essential to overcome the above issues. To design efficient MOs catalysts, it is critical and necessary to understand the adsorption mechanism and associated catalytic processes of LiPSs. However, most reviews still lack a comprehensive investigation of the basic mechanism and always ignore their in‐depth relationship. In this review, a systematic analysis toward understanding the underlying adsorption and catalytic mechanism in Li—S chemistry as well as discussion of the typical works concerning MOs electrocatalysts are provided. Moreover, to improve the sluggish “adsorption‐diffusion‐conversion” process caused by the low conductive nature of MOs, oxygen vacancies and heterostructure engineering are elucidated as the two most effective strategies. The challenges and prospects of MOs electrocatalysts are also provided in the last section. The authors hope this review will provide instructive guidance to design effective catalyst materials and explore practical possibilities for the commercialization of Li—S batteries.

## Graphical abstract

Metal oxides are promising electrocatalysts in lithium‐sulfur (Li—S) batteries for practical commercializations. In this review, the basic mechanisms and rational models are proposed to understand the adsorption and catalytic behaviors in Li—S system. Based on these models, two effective strategies concerning the defect fabrication to overcome the low conductivity of metal oxides are concluded.

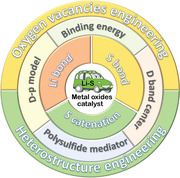

## 1 Introduction

Standing at the gateway to a new era, we have witnessed dramatic changes of energy storage technologies over recent decades, especially the appearance of electric vehicles (EVs) on roads indicates a first step toward a green and smart society. Since the first successful commercialization of the lithium ion (Li‐ion) battery by SONY, Li‐ion batteries have dominated the energy storage market for over 30 years, greatly impacting to the society and our daily lives. However, each energy storage technology is limited by its theoretical capacity. The quest for a “beyond Li‐ion battery” system is thus ongoing.

**Figure 1:**
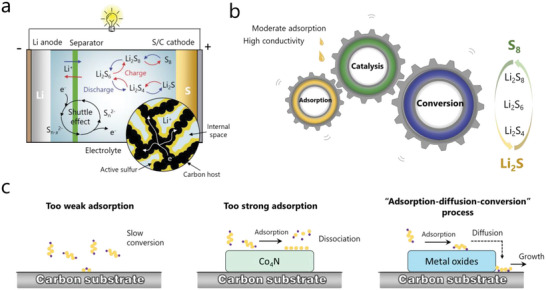
a) Illustration of charge transportation and reversible reaction in Li—S battery. b) The function and basic mechanism of adsorption, catalysis, and conversion. c) Polysulfide behaviors of carbon substrate, Co_4_N catalyst, and metal oxide catalyst, respectively.

Among the competitors in the high energy storage arena, lithium sulfur (Li—S) batteries emerged as a promising system with a high theoretical specific capacity of 1675 mAh g^−1^ and potential energy density up to 2600 Wh kg^−1^, which is more than 5 times of the value of Li‐ion batteries. In addition with the substantially reduced costs and intrinsic environmental benignity of Li—S chemistry, these superiorities make Li—S chemistry an attractive candidate for the application in smart grid and EVs.^[^ ^1^ ^]^ Nevertheless, the development of Li—S batteries is hampered by a series of fundamental challenges. First, the inherently insulating nature of S_8_ and reduced component Li_2_S, with low conductivities of 5 × 10^−30^ and 10^−13^ S cm^−1^, respectively, impedes the transfer of electrons. As such, conductive additives are added to improve cathode electron conductivity. Second, the volume change between S_8_ and Li_2_S is nearly 80%, where the volume strain leads to negative effects in the durability of Li—S batteries. Last but most important, the soluble intermediate lithium polysulfides (LiPSs) dissolve in commonly used electrolytes and travel between cathode and anode driven by concentration diffusion, which is generally known as “shuttle effect”. This is the main reason for the low columbic efficiency and rapid capacity fading.^[^ ^2^ ^]^ Moreover, LiPSs shuttling can also result in fast corrosion of the Li anode to lead to an increased cell impedance, which further hampers the batteries’ development.^[^ ^3^ ^]^ The reaction mechanism is well illustrated in **Figure** **1**a.

Confining active sulfur into porous conductive carbonaceous material was the main approach in the early stages of development.^[^ ^1^, ^2^, ^4^ ^]^ In this strategy, the LiPSs' escape and shuttling is suppressed by physical spatial confinement, and the sulfur expansion is also accommodated by internal space, which can result in a significant improvement in cycling stability. Examples of carbonaceous hosts include mesoporous carbon, carbon nanotubes (CNTs), carbon nanocages, hierarchical porous carbon, and so on.^[^ ^5^ ^]^ These carbonaceous materials, which act as sulfur host, require high conductivity with large surface area and porous structure.^[^ ^6^ ^]^ However, a host material with excess porosity and surface area will also lead to negative effects: 1) resulting in a low tapped density cathode which needs large amounts of electrolyte for infiltration; and 2) leading to the formation of excessive cathode electrolyte interface (CEI) and consuming extra electrolyte, since the amount of electrolyte is critical to the pack energy density of batteries.^[^ ^7^ ^]^ Therefore, cell parameters such as sulfur loading, tapped density and E/S (electrolyte/sulfur) ratio need to be paid attention to, which is of significance to commercial applications.^[^ ^8^ ^]^ In the past few years, we have witnessed Li—S batteries with prominent capacity and superb cyclic performances even at severe environmental conditions enabled by catalytic materials as cathode host or separator interlayer.^[^ ^9^ ^]^ The interfacial chemistry–chemical adsorption and catalytic conversion, play the key role to achieving such high cell performances.

Chemical adsorption between adsorption sites and LiPSs can not only anchor polysulfide to alleviate LiPSs mitigation, more importantly, the strong adsorption can also alter the reaction path of LiPSs, thus accelerating the formation of products, resulting in a catalytic conversion. As catalysis is very important in Li—S chemistry, it can greatly decrease the activation energy with a minor amount of catalyst to improve the sulfur utilization. Meanwhile, it can prevent LiPSs from being accumulated, thus further suppressing the shuttle effect.^[^ ^10^ ^]^ Till now, numerous adsorbents have been developed, such as functional groups, heteroatoms, metal compounds, black phosphorus and so forth, with different adsorption strength and mechanism.^[^ ^11^ ^]^ Notably, a stronger adsorption does not necessarily correspond to a better catalytic property. Said otherwise, a too strong adsorption can dissociate the original Li—S bond of LiPSs and impedes the LiPSs formation, which could reduce the conversion kinetics. For example, density functional theory (DFT) calculations revealed that the interaction of Li_2_S_6_ on Co_4_N is so strong that the Li—S bond will be broken, resulting in a sulfurized surface of Co_4_N similar to CoS_2_ (Figure 1c).^[^ ^12^ ^]^ Therefore, understanding the adsorption mechanism and screening the optimum binding configurations, as well as deciphering the catalytic activity, are of great importance for effective catalyst material design. Today theoretical models provide valuable insights for the smart design of catalysts and can speed up the search for good materials.^[^ ^13^ ^]^ Although recent reviews are available in summarizing those adsorbents and catalytic materials, as well as their synergies, little attention was paid to unraveling basic principles and the internal relationships between adsorption and catalytic property.

Metal oxides (MOs) that combine high polarity, chemical stability and low cost emerge as the most promising catalytic materials in Li—S systems, and which are considered to provide moderate adsorption to polysulfide. So far, many MOs like TiO_2_, MnO_2_, Fe_3_O_4_, Co_3_O_4_, CeO_2,_ etc. were found to possess efficient adsorption and catalytic activities in Li—S chemistry.^[^ ^2^, ^14^ ^]^ However, the inherent poor electrical conductivity nature of MOs is unfavorable for electron transfer and will impede the direct LiPSs conversion on its surface. Thus, an additional diffusion step is generally needed for LiPSs to migrate to the ternary interface between electrolyte, insulating MOs and conductive substrate, resulting in the “adsorption‐diffusion‐conversion” process (Figure 1c).^[^ ^15^ ^]^ Unlike some high conductive catalysts (i.e., metal carbides or nitrides) where adsorption and catalytic conversion can proceed directly on their surface, the extra diffusion step on low conductive MOs requires extra energy, which slows down the reaction kinetics.^[^ ^15a^ ^]^ This is a main reason for the limited catalytic effectiveness of low conductive MOs. To overcome this inherent problem, two promising strategies are concluded from the recent literature. One is to introduce oxygen vacancies (OVs). It is reported that oxygen vacancies in metal oxides enhance the intrinsic electron conductivity as well as catalytic activity. OV‐rich MOs with altered electron structure manifest conductive properties and can basically change the catalytic process.^[^ ^16^ ^]^ The other one is constructing heterostructures. Unlike improving the intrinsic conductivity of MOs, introducing heterostructures can smoothen the “adsorption‐diffusion‐conversion” process.^[^ ^15b^ ^]^ Moreover, the generated heterojunction can show a higher catalytic ability toward LiPSs conversion.^[^ ^17^ ^]^ With oxygen vacancies or heterostructures, intriguing performances have been achieved for MOs composite based Li—S batteries.^[^ ^18^ ^]^ However, so far, a review that summarizes these two modifications to overcome the insulating properties for achieving high catalytic activity composite materials is still missing.

To fill this gap and figure out the principles, a thorough investigation of MOs electrocatalysts is needed. In this review, we first aim to excavate the underlying mechanism by generalizing different types of interactions between LiPSs and adsorbent and summarize recently developed models and descriptors to evaluate the catalytic activities. Recent works on various MOs’ electrocatalysts in Li—S batteries are subsequently reviewed, in which MOs are employed in either cathode host or separator interlayer. Finally, by combining above catalytic models, MOs with oxygen vacancies and heterostructure engineering are comprehensively elaborated and reviewed with respect to their perspectives. As a whole, we hope this review offers instructive guidance to explore effective metal oxide‐based conductive materials with superior adsorption–catalytic properties for the commercialization of Li—S batteries.

## 2 Mechanism of Adsorption and Catalysis in Li‐S Chemistry

### 2.1 Adsorption

As we know, the dissolution of intermediate LiPSs into the organic electrolyte causes the “shuttle effect” of LiPSs, leading to capacity fading and low Coulombic efficiency. It should be pointed out that the dissolution is determined by the intermolecular attraction between LiPSs and electrolyte solvent.^[^ ^19^ ^]^ Therefore, a stronger attraction from the host is necessary to suppress the dissolution of LiPSs and further realize the suppression of the shuttle effect.

**Figure 2:**
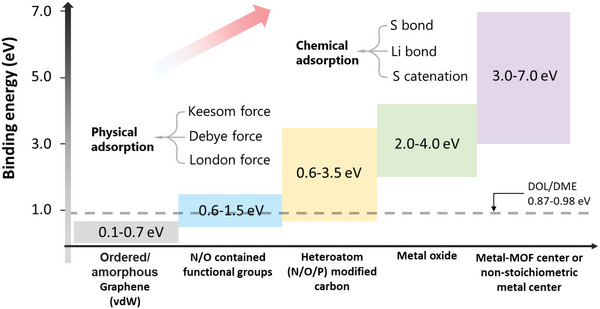
The interactions scope of categorized adsorbents to LiPSs. The binding energies are derived from computations.

We start the discussion with the interaction between the electrolyte (DOL/DME) and soluble LiPSs (Li_2_S*_x_*, 4 ≤ *x* ≤ 8). DFT theory was used to calculate the binding energy value of DOL and DME, which is about 0.87–0.98 eV.^[^ ^20^ ^]^ To realize an adsorption effect, the interaction between host and soluble LiPSs should be stronger than this value. When graphene was employed as sulfur host, the binding energy between graphene and LiPSs was calculated as 0.1–0.4 eV, which is smaller than that of the electrolyte.^[^ ^21^ ^]^ This is the main reason for massive LiPSs dissolution and the shuttle effect in bare carbon hosts. In order to enhance the adsorption between LiPSs and the conductive host, fabricating polar surface has been proven to be quite effective. As shown in **Figure** **2**, multiple kinds of polar sites have attracted attention like functional groups, heteroatoms (N/O/P etc.), metal based compounds, metal‐organic framework (MOF) based composites and so forth, rendering much stronger interactions toward LiPSs.^[^ ^22^ ^]^ According to bond strength, these interactions can be divided into physical adsorption and chemical adsorption. The physical adsorption, which is caused by van der Waals (vdW) forces, combines Keesom orientation forces (*F*_o_), Debye induction forces (*F*_I_) and London dispersion forces (*F*_D_), representing interactions between polar–polar, polar–nonpolar and nonpolar–nonpolar species, respectively.^[^ ^23^ ^]^ The interaction between graphene and LiPSs is of this type. However, due to the weak binding characteristic, the physical adsorption is not strong enough to realize stable anchoring of LiPSs. For a strong binding, chemical adsorption is required, which is also the key in the following adsorption study. According to different function principles, we classify these chemical adsorptions into the following categories: Li bond, S bond and sulfur‐chain catenation. In this part, the Li bond and S bond will be first introduced below.

#### 2.1.1 Li Bond

**Figure 3:**
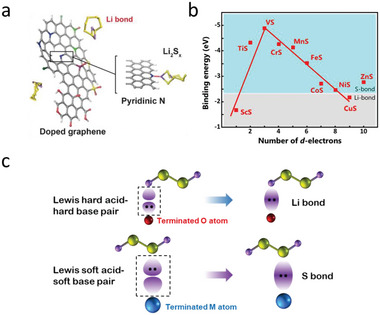
a) Schematic diagram of Li bond in pyridinic N. Reproduced with permission.^[^ ^24b^ ^]^ Copyright 2017, Wiley VCH. b) Periodic law of binding energy among first‐row TMS. Reproduced with permission,^[^ ^28^ ^]^ Copyright 2017, American Chemical Society. c) Schematic diagram of the formation of Li bond and S bond via Lewis acid–base pairs using Li_2_S_2_ as example.

The Li bond is formed between the electronegative polar site and terminal Li atom in LiPSs, which can be presented as Li–X (X represent O, N, P, etc.).^[^ ^21^, ^24^ ^]^ A typical example is the Li‐pyridinic N (pN) interaction in N‐doped graphene (NG) (**Figure** **3**a). Such interaction is considered as a kind of dipole–dipole interaction.^[^ ^24b^ ^]^ It is worth noting that the Li bond must be distinguished from Keesom interactions, as the Li bond showcases a much higher dipole moment and bond strength. Certainly, the Li bond should also be differentiated from traditional ionic bonds for the much higher binding of the ionic bond. Meanwhile, the formation of an ionic bond is always accompanied by charge transfer, while charge transfer in the Li bond is negligible. Therefore, the Li bond can be defined as a unique bond originating from electrostatic attraction with a binding energy between Keesom interaction and ionic bond.^[^ ^25^ ^]^

Lewis acid–base theory was proposed to explain the formation of Li bonds. In a typical Li–pN interaction, the pN with extra lone pair of electrons in the *p*‐orbitals can act as Lewis base, while terminal Li in LiPSs with vacant orbital serves as Lewis acid. As a result, a relatively strong binding is obtained by Lewis acid–base coordination. This theory also explains that only pN in NG can form a Li bond with LiPSs. For other two kinds of N (i.e., pyrrolic N and graphitic N) without lone pair electrons in their valance orbitals, the interaction between them and LiPSs are simply identified as normal Keesom interaction. Therefore, it is concluded that the fundamental difference between the Li bond and Keesom interaction lies in the availability of lone pair electrons.^[^ ^24^, ^26^ ^]^ In addition, based on HSAB (Hard–Soft–Acid–Base) theory, terminated Li atoms with high positive charge and small size can be considered as hard acid. Thus, Li atoms easily form bonds with a hard base possessing a similar large negative charge and small size atom, which are typically polarized X (O, N, P, etc.) atoms.^[^ ^27^ ^]^ This theory provides a new perspective to explain the formation tendency of Li bonds.

#### 2.1.2 S Bond

Except terminal Li that act as hard Lewis acid in Li‐bonding, the polysulfide anions (S*_x_* ^2−^) can also render it as a soft Lewis base for the lone electron pairs of sulfur. Therefore, in principle, host materials exhibiting soft Lewis acid character can also strongly interact with LiPSs to show Lewis acid–base interaction, such bond is generally called S bond. Different from the Li bond that is mainly based on a Coulombic effect, the S bond has a more covalent nature.^[^ ^27^, ^28^ ^]^ In this regard, transition metal (TM) composites possessing vacant *d* orbitals are capable to serve as soft Lewis acid to coordinate with S*_x_* ^2−^ to form TM‐S bonds. To investigate the effect of d‐orbital filling on the interaction, theoretical calculations for various TM‐S configurations among first‐row transition metal sulfides were conducted. An analogous periodic law for S bond was subsequently proposed. As shown in Figure 3b, from ScS to ZnS, there are two nearly linear scaling lines where VS lies on the top indicating the strongest binding. Such a volcano‐type correlation is the result of the counterbalance between valence electron number and unoccupied *d*‐orbital number. This periodic law provides valuable guidance for screening TM and targeting strong adsorption to restrain the shuttle effect.^[^ ^28^ ^]^

For transition metal oxides (TMOs), it can be considered that Li bond and S bond coexist. In most cases, the Li bond dominates the binding energy as most TMOs are oxygen terminated. But in the case of sub‐stoichiometric oxides or unsaturated metal, the S bond also contributes significantly to the interaction.^[^ ^21^, ^28^ ^]^ Material that is able to form a Li bond or S bond with LiPSs correspondingly exhibit lithiophilic or sulfiphilic behavior, respectively.

To note here, instead of previous classified polar–polar interaction and Lewis acid–base interaction,^[^ ^21^, ^29^ ^]^ we describe chemical adsorption between host and LiPSs as Li bond and S bond, which is more precise to understand the diverse adsorption behaviors. These two bonds are considered as Lewis acid–base interaction. As shown in Figure 3c, both bonds originate from the coordination between filled orbitals and vacant orbitals, which are served as Lewis base and Lewis acid, respectively. The HSAB theory can better explain the diverse behaviors of LiPSs, where terminal Li atom and S*_x_* cluster in polysulfide (Li—S*_x_*—Li, 2 < *x* < 8) can be considered as hard acid and soft base, providing vacant orbital and lone electron pairs, respectively. In addition, as the adsorption is specified into orbital hybridization between adsorbents and LiPSs, we can correlate the bond properties to the electron's occupancy and antibonding state in hybridization orbitals, which provides a new method in catalytic research.^[^ ^30^ ^]^

### 2.2 Catalysis

In addition to trapping polysulfide via binding to prevent dissolution and shuttling, the reaction kinetics also plays a significant role. Sluggish kinetics always leads to the accumulation of numerous LiPSs at the cathode and inevitably result in LiPSs diffusion. Moreover, arbitrary precipitation is also possible due to the sluggish kinetics, resulting in the formation of large Li_2_S particles that are difficult to be reused. Therefore, materials with high catalytic capability can effectively enhance the reaction kinetics which can contribute to suppressing the shuttle effect and ensure uniform precipitation, leading to a high sulfur utilization. In addition, it can also reduce the overpotential and improve the high rate performance. In this part, the basic principles and underlying mechanisms for catalysis are discussed and presented for lithium sulfur chemistry, which is vital for screening high‐activity catalysts.

#### 2.2.1 Rate‐Determining Step of Li‐S Chemistry in Discharge Process

**Figure 4:**
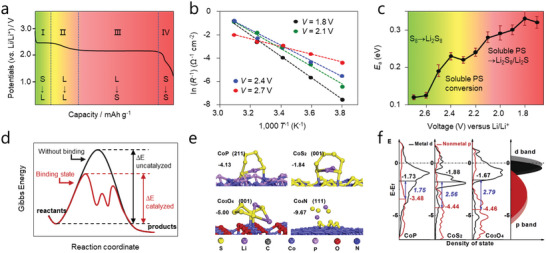
a) Voltage profile of discharge process of Li—S cell. b) Arrhenius plots of the line relationship of charge transfer resistance and temperatures from 1.8–2.1 V. c) Activation energy profiles at different voltages. Reproduced with permission.^[^ ^40^ ^]^ Copyright 2021, Wiley VCH. d) Alteration of reaction pathway for binding configuration. e) Binding energies and adsorption configurations of CoP, CoS_2_, Co_3_O_4_, and Co_4_N to Li_2_S_6_. f) DOS analysis of d and p band for of CoP, CoS_2_, and Co_3_O_4_, respectively. Reproduced with permission.^[^ ^12^ ^]^ Copyright 2018, Elsevier.

Sulfur reduction during discharging undergoes a complex multistep electrochemical conversion reaction, which can be divided into four steps marked with four regions in **Figure** **4**a. Step I is a solid–liquid transition in which the initial S_8_ reacts with Li^+^ to form Li_2_S_8_. Step II is a liquid–liquid transition in which dissolved Li_2_S_8_ reduces to Li_2_S_6_ and finally to Li_2_S_4_. Step III is a liquid‐solid transition for the dissolved Li_2_S_4_ to insoluble Li_2_S_2_ or Li_2_S. Noting that Li_2_S_2_ and Li_2_S coexist, step IV is a solid–solid transition in which the insoluble Li_2_S_2_ further transfers to insoluble Li_2_S. In particular, due to the non‐conductive nature of both Li_2_S_2_ and Li_2_S, the final step suffers from very high polarization,^[^ ^31^ ^]^ making the region IV negligible in most cases. Therefore, the whole process during discharging can be considered as the three processes solid–liquid dissolution, liquid–liquid transformation, and liquid–solid precipitation.

To figure out the rate‐determining step, the activation energies (*E*_a_) for each step were determined experimentally based on the Arrhenius equation. As shown in Figure 4c, the charge transfer resistance (*R*_ct_) at each voltage at various temperatures (Figure 4b,c) was investigated via impedance techniques.^[^ ^32^ ^]^ It was found that the rate‐determining step is the final precipitation process, which controls the kinetics of reaction and is responsible for the shuttle effect. Thus, focus should be paid on the precipitation steps when considering the electrocatalytic reaction in Li—S chemistry.

#### 2.2.2 Principles of Li‐S Catalysis and *D*‐Band Theory

In Li—S batteries, catalysis which enables fast conversion kinetics by lowering the energy barriers through active sites can be evidenced by some experimental results related to specific electrochemical parameters such as charge transfer impedance, overpotential, Tafel plot, and so on.^[^ ^33^ ^]^ However, the intrinsic catalytic activity on each active site and their functions are difficult to be identified and understood, as the catalytic activity is not directly correlated with the above experimental parameters, where the sites numbers or host structures also heavily influence these experimental results. The mechanism of the catalysis still remains unclear. Therefore, understanding the root of catalytic effect in Li—S batteries is of great significance. In addition, besides previous screening of different catalysts which relied on complex trial‐and‐error experiments, plenty of high‐throughput calculations, and machine learning have been implemented to unfold the mysteries of LiPSs catalytic conversion. In this strategy, simple and easily obtainable physical/chemical properties termed reactivity descriptors were proposed to correlate with reaction energies or activation barriers. Thus, a scaling relationship between descriptors and catalytic activity is established, which is meaningful for the exploration of fundamental catalytic mechanisms and screening high catalytic electrocatalysts.^[^ ^34^ ^]^

As is well documented, the catalytic process of Li—S chemistry is a heterogeneous catalysis. Different from many gas‐phase catalytic processes that follow the Eley–Rideal pathway, the Langmuir–Hinshelwood mechanism applies more to the Li—S catalysis with a solid phase product. Thus, the catalytic process toward LiPSs consists of the following steps: the adsorption of LiPSs, diffusion and reaction at the active sites and finally desorption of the product from the catalyst surface. These steps involve the exchange of Li ions and electrons, with the bond breaking and formation between reactant and catalytic active sites.^[^ ^34^, ^35^ ^]^ From this point of view, binding energy which can well describe the bond strength is considered as a descriptor in Li—S catalysis. In a typical adsorption–catalytic process, a strong binding between active site and LiPSs will weaken the S—S bond in the sulfur chain, which makes high order LiPSs easier to be dissociated and converted into low order sulfur chains, effectively promoting LiPSs reduction (Figure 4d). However, too strong interaction with the stable configuration may also impede the transformation and block the surface reaction sites. This phenomenon has been elucidated as “Sabatier principle”, where binding energies and catalytic activities are plotted as a “volcano‐type” function.^[^ ^36^ ^]^ Thus, Sabatier principle could be a qualitative way to predict the activity of catalysts. To further understand the complex processes, a molecular‐level study is needed to be established.

The *d*‐band theory proposed by Hammer and Norskov aids the understanding of the bond formation and catalytic activity.^[^ ^37^ ^]^ In the *d*‐band theory, the *d* band of TMs plays a major role in the interaction with LiPSs, and the band of *d*‐state can be approximately considered as a single energy state called *d* band center (*ε*_d_). Thus, a stronger adsorption to the LiPSs can be correlated to the up shifting of *d*‐band center toward Fermi energy, for the up shifting of *d*‐band would make antibonding states arising and probably be emptied, thus resulting in an enhanced interaction.^[^ ^38^ ^]^ Therefore, the *d*‐band center can be considered as a descriptor, where *ε*_d_ shifts toward the Fermi level corresponding to a strong interaction. When using the *d*‐band center as descriptor, different modulations like doping, alloying, strain, etc. that can alter the catalytic properties can be well explained. For example, when alloying metallic Ni with Fe, the coordination number of Ni would be reduced, resulting in the upshift of the *d*‐band center toward Fermi level. As a consequence, it brings an enhanced kinetics with a high electrocatalytic activity.^[^ ^39^ ^]^ The *d*‐band center has also been validated to be an efficient descriptor for many catalytic processes with transition metal catalysts.^[^ ^34b^ ^]^

#### 2.2.3 *D*‐*p* Model for TM Compound

In *d*‐band theory, the metal centers are usually regarded as active sites for the redox reactions and the position of *d*‐band center toward Fermi level is responsible for the catalytic activity. However, this descriptor is not always correct especially in TM compounds. For example, Qian et al. investigated the catalytic properties of various Co based compounds (CoP, CoS_2_, Co_3_O_4_, and Co_4_N).^[^ ^12^ ^]^ As shown in Figure 4e,f, compared with CoP and CoS_2_, Co_3_O_4_ exhibits both a stronger binding energy and smaller gap between *d*‐band center and Fermi level. However, experimental studies showed that CoP possesses the most superior performances as well as the highest catalytic activity rather than Co_3_O_4_. To explain this, the authors proposed the *p*‐band center of anions, *p* orbitals of catalysts, and ascribed the higher catalytic activity of CoP to the narrower gap between *p*‐band center of anions and d‐band center of metals. As shown in Figure 4f, compared with CoS_2_ and Co_3_O_4_, the *p*‐band center of CoP shifts closer toward the Fermi level, thus resulting in a narrower energy gap between 3*d* and 2*p* band center. The softer and less electron pulling character of the P atom would increase the energy of bonding state and reduce the energy gap between bonding and anti‐bonding orbitals, thus presenting a higher degree of hybridization and contribution to valance electron energy, facilitating the electron exchange and redox dynamics. Meanwhile, a similar work to compare the catalytic activity of Fe based oxide and phosphide compounds was performed by Zhu's group.^[^ ^41^ ^]^ They obtained the same conclusion as Qian's work that the higher the shift of the *p*‐band center in FeP, the higher the contribution to catalytic activity. Other analogous works also support that a smaller energy band gap between the *d* and *p* bands in TM compounds corresponds to a better catalytic activity, which are all well in agreement with the experimental values.^[^ ^42^ ^]^ From these cases, Δ*ε* (*p*–*d*) can predict the catalytic activities and can thus also be a good descriptor in TM compounds. Based on this, the *d*–*p* model was subsequently proposed, which provides valuable guidance for rationally screening TM based compound catalysts.

Although the importance of the catalytic effect is gradually being appreciated in Li—S research, the catalysis in Li—S chemistry cannot be considered as a traditional heterogenous catalysis with gas‐phase reactants and product. It should be noted that desorption of solid Li_2_S_2_/Li_2_S is difficult, which would poison the active sites and lead to progressive catalyst deactivation. There are some works that have provided solutions to address the accumulation of S/Li_2_S_2_/Li_2_S. For example, an electrolyte strategy was proposed to realize a full dissolution of Li_2_S*_x_* (1 ≤ *x* ≤ 8) and transfer the solid related reactions into liquid–liquid reactions.^[^ ^43^ ^]^ Inspired by this, the concept of “surface cleaning electrolyte additives” was also proposed to sweep and refresh the catalyst surface by dissolving the solid deposits.^[^ ^44^ ^]^ These strategies are intended to dissolve Li_2_S into electrolyte to maintain the activities of electrocatalysts. In addition, except heterogeneous electrocatalysis, the homogeneous electrocatalysis was also introduced to improve the reaction kinetics in Li—S batteries. These homogenous electrocatalysts are a kind of soluble small‐molecules additives, which are usually called redox mediators (RMs).^[^ ^15^, ^45^ ^]^ In this respect, the RMs enables the full exposure to LiPSs and serve as accelerator to promote the reaction conversion by generating additional pathways. And the coverage of active sites and the degraded activities can be avoided by this strategy. Nevertheless, highly effective catalysts in heterogenous catalysis are still effective in promoting electrochemical performances by enhancing conversion between soluble polysulfides and facilitating the precipitation and dissolution of solid Li_2_S_2_/Li_2_S.^[^ ^46^ ^]^

### 2.3 Thiosulfate/Polysulfide pathway

#### 2.3.1 Sulfur‐Chain Catenation

Except for the abovementioned Li bond and S bond mechanism that relied on the surface affinity between host material and LiPSs, another and different chemical approach can also entrap LiPSs by reversibly catenating and grafting polysulfide chains into a kind of sulfur contained complex, a process called sulfur catenation. For example, as a member of promising all solid state battery materials, lithium thiophosphate (Li_3_PS_4_) can reversibly react with sulfur to yield a family of sulfur‐rich lithium polysulfidophosphates (LiPS_4+_ *_n_*) with equally high ionic conductivity.^[^ ^47^ ^]^ In this process, sulfur atoms were reversibly grafted into PS_4_^3−^ through breaking and formation of S—S bonds. Based on this mechanism, Nazar et al. found a kind of thiosulfate groups (S_2_O_3_^2−^) that can also reversibly catenate polysulfide to form a polythionate complex (O_3_S—S*_x_*—S).^[^ ^48^ ^]^ Specifically, during the discharge process in the Li—S cell, the host such as manganese dioxide can serve as prototype reacting with the initially formed LiPSs to generate the initial thiosulfates. The formed thiosulfate species thereby anchor LiPSs by catenating them through the S—S bond to form surface‐bonded intermediates polythionate complexes, and finally convert them into Li_2_S via disproportionation (Equation (1)). This new trap mechanism on the surface of MnO_2_ with thiosulfate–polythionate conversion has its basics in the so‐called “Wackenroder reaction” reported over a century ago.^[^ ^49^ ^]^ Apart from MnO_2_, graphene oxide (GO) and MXene was also proposed to function in the same manner.^[^ ^48^, ^50^ ^]^ This catenation mechanism is widely existent in Li—S chemistry.^[^ ^9^, ^51^ ^]^

(1) $$\begin{array}{ccc} 2{\left[O-\begin{array}{c} \begin{array}{c} O\\ ∥ \end{array}\\ S\\ \begin{array}{c} ∥\\ O \end{array} \end{array}-S\right]}^{2-}+S_{X}^{2-} & ⇄ & {\left[O-\begin{array}{c} \begin{array}{c} O\\ ∥ \end{array}\\ S\\ \begin{array}{c} ∥\\ O \end{array} \end{array}-S-{\left(S\right)}_{X-2}-S-\begin{array}{c} \begin{array}{c} O\\ ∥ \end{array}\\ S\\ \begin{array}{c} ∥\\ O \end{array} \end{array}-O\right]}^{2-}\\ & & +\;2S^{2-}\left(X>3\right) \end{array}$$

#### 2.3.2 Polysulfide Mediator

**Figure 5:**
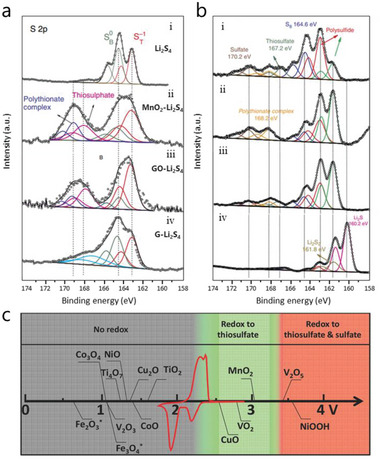
a) The S 2*p* spectrum of i) Li_2_S_4_, ii) MnO_2_‐Li_2_S_4_, iii) GO‐Li_2_S_4_ and iv) graphene‐Li_2_S_4_. Reproduced with permission.^[^ ^14^ ^]^ Copyright 2017, Wiley‐VCH. b) The S 2*p* spectrum of S/MnO_2_ nanosheet electrodes i) discharged to 2.15 V, ii) discharged to 2.15 V and aged 20 h, iii) discharged to 800 mAh g^−1^ and iv) discharged to 800 mAh g^−1^ and aged for 20 h. All cells were charged at 0.05 C. Reproduced with permission.^[^ ^53^ ^]^ Copyright 2021, Wiley‐VCH. c) Chemical reactive window for different metal oxides versus Li/Li^+^. Reproduced with permission.^[^ ^51a^ ^]^ Copyright 2016, Wiley‐VCH.

As LiPSs can be reversibly catenated and grafted into a kind of thiosulfate/polythionate species through a sulfur‐chain catenation mechanism, it is worth noting that the formed thiosulfate/polythionate can act as polysulfide mediator (endogenous RMs) to further catalyze the conversion of LiPSs.^[^ ^48^ ^]^ As shown in **Figure** **5**a, when different hosts (MnO_2_, GO, and Graphene) were immersed in Li_2_S_4_ solution, it was found that thiosulfate/polythionate active groups are generated on both surfaces of the MnO_2_ and GO host, implying that the polysulfide was initially oxidized into a thiosulfate and polythionate complex. Moreover, for the MnO_2_/S electrode with a discharge state of 2.15 V, after aging for 20 h (Figures 5b‐i and 5‐ii), the peaks of thiosulfate have disappeared but the polythionate complex increased. Besides, the long‐chain LiPSs was transformed to shorter‐chain LiPSs, as evidenced by the change in the ratio of terminated S (green, S_T_^−1^) and bridging S (red, S_B_^0^) peaks. Thus, we conclude that the thiosulfate is able to react with longer‐chain LiPSs species to yield shorter LiPSs and polythionate. This process can be considered as a new method to alter the regular reaction pathway of LiPSs.^[^ ^51^, ^52^ ^]^

However, it should be noted that not all MOs can trigger the initial formation of thiosulfate or catalyze LiPSs conversion in the same manner.^[^ ^51a^ ^]^ As shown in Figure 5c, different MOs are distinguished by their chemical reaction window versus Li/Li^+^. The reversible thiosulfate formation can only be triggered in the redox potential window between 2.4–3.05 V such as with VO_2_ and MnO_2_. The reversible formation is evidenced in Figure 5b‐iii,iv, where both thiosulfate and polythionate were consumed at the end of discharging, while the MOs with values below that window such as TiO_2_ and Fe_2_O_3_ cannot trigger the reaction. In this case, there is only Li bond or S bond formation but thiosulfate/polythionate transformation would not participate in the Li—S reaction. Other MOs such as V_2_O_5_ with values lying above the window would give rise to over‐oxidization of inactive sulfate groups which would result in inferior performances. For these materials lying above the window, an adjusted cycle voltage was recommended to avoid the formation of inactive sulfate species aiming to realize a long cycle life. In short, this so called “Goldilocks” principle provides valuable insights into the correlation of fundamental surface mechanism and cell stability in a thiosulfate/polysulfide pathway, which is essential to design rational MOs for high performance Li—S batteries.

## 3 Metal Oxides (MOs) Based Conductive Materials

**Figure 6:**
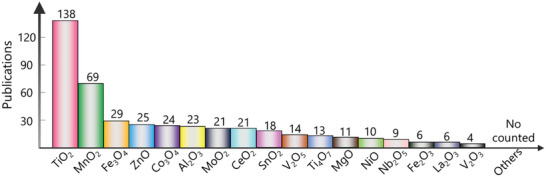
The numbers of publications related to Li—S adsorption or catalysis for various metal oxides.

The semiconducting metal oxides (MOs) always exhibit strong polar surfaces derived from high electronegative oxygen terminated surfaces. The exposed anionic surface O^2−^ will have Lewis base character and be thought of possessing lone pair electrons which are responsible for forming a Li bond with LiPSs, giving it potential for providing moderate adsorption and efficient catalytic conversion in Li—S chemistry.^[^ ^54^ ^]^ Compared with other metal compounds like metal carbides, sulfides, nitrides and phosphides, MOs can provide a much more stable catalytic surface at a lower cost. However, due to their insulating nature, MOs are not preferable for acting as sulfur hosts. But they could serve as effective additives integrating with carbonaceous materials to fabricate composite hosts by combining both polar adsorption of MOs and conductivity of carbonaceous materials with both electron pathways and ion diffusion channels. For example, Al_2_O_3_,^[^ ^55^ ^]^ SiO_2_,^[^ ^56^ ^]^ Mg_0.6_Ni_0.4_O^[^ ^57^ ^]^ and TiO_2_^[^ ^58^ ^]^ nanoparticles were incorporated in a conductive carbon matrix through mechanical mixing, which delivered improved capacities and cycling stabilities as well as Coulombic efficiencies compared to a bare carbon host or MOs host. Besides mechanical milling, hydrothermal or heat treatment of precursors is a more common strategy to synthesize composites with better integration of MOs and carbon.^[^ ^59^ ^]^ In addition, the high adsorption and catalytic effect of MOs was reported to potentially overcome the reliance on LiNO_3_ additives, which contributes to a safer Li—S battery. For example, Ding et al. used a RuO_2_ catalyst in the sulfur cathode, equipped it with the LiNO_3_‐free cells and achieved higher capacity and improved capacity retention as compared to their LiNO_3_‐based counterpart.^[^ ^60^ ^]^ **Figure** **6** showcases the publication numbers of MOs studied in Li—S batteries in the last decade, showing that TiO_2_ and MnO_2_ are the most widely studied MOs. **Table** **1** summarizes the reported works for MOs based conductive materials employed as cathode and separator interlayer. We note that MOs as cathode host and interlayer are intrinsically the same, in which MOs are equivalently responsible for accelerating transfer conversion and Li_2_S precipitation. In the following section, some typical MOs (i.e., TiO_2_, Ti_4_O_7_, MnO_2_, Fe_3_O_4_, Co_3_O_4_, MoO_2_, CeO_2_, and SnO_2_) will be briefly discussed.

**Table 1:** Examples of performances of different MO*_x_* based conductive materials

MO*_x_* based conductive materials	Application	Cathode area loading (sulfur content)/separator thickness	Electrolyte/sulfur (E/S) ratio	Capacity retention (rate, cycles)	Capacity decay per cycle	Ref.
G/TiO_2_	Cathode	1.5–2 mg cm^−2^ (60%)	NA	737 mAh g^−1^ (0.5 C, 100 cycles)	0.25%	[61]
CP@TiO_2_	Cathode	2 mg cm^−2^ (40%)	NA	850 mAh g^−1^ (0.5 C 200 cycles)	0.24%	[62]
G/TiO_2_	Separator	0.51 mg cm^−2^ (51.2%)/3 µm	NA	1040 mAh g^−1^ (0.5 C, 300 cycles)	0.00003%	[63]
HCNF@TiO_2_	Cathode	2 mg cm^−2^ (70%)	NA	380 mAh g^−1^ (1 C, 500 cycles)	0.12%	[64]
TiO_2_/G/NPCDs	Cathode	1.2 mg cm^−2^ (55%)	NA	618 mAh g^−1^ (1C, 500 cycles)	0.074%	[59a]
MH‐SiO_2_@TiO_2_	Cathode	NA (80%)	13 µL mg^−1^	264 mAh g^−1^ (1 C, 1000 cycles)	0.066%	[65]
CNT‐T2@TiO_2_	Separator	1.7 mg cm^−2^ (60%)/6 µm	NA	803 mAh g^−1^ (0.1 C, 200 cycles)	0.25%	[66]
C@TiO_2_@C	Cathode	2.5 mg cm^−2^ (76.4%)	NA	511 mAh g^−1^ (2 C, 500 cycles)	0.068%	[67]
SDC@TiO_2_	Cathode	2 mg cm^−2^ (60%)	NA	569 mAh g^−1^ (1.5 A g^−1^, 1500 cycles)	0.024%	[68]
MWCNTs@TiO_2_ QDs	Separator	0.8 mg cm^−2^ (60%)/5 µm	NA	610 mAh g^−1^ (0.5 C, 600 cycles)	0.073%	[69]
C—Co/TiO_2_	Cathode	1.5 mg cm^−2^ (70%)	NA	466 mAh g^−1^ (1 C, 300 cycles)	0.15%	[70]
CNT@TiO_2−_ *_x_*	Cathode	2.2 mg cm^−2^ (72.9%)	12 µL mg^−1^	590 mAh g^−1^ (1 C, 500 cycles)	0.053%	[16]
TiO_2_‐MXene	Separator	1.2 mg cm^−2^ (70%)/5 µm	NA	576 mAh g^−1^ (2 C, 1000 cycles)	0.028%	[71]
TiO—TiO_2_/PPy	Cathode	1 mg cm^−2^ (75%)	30 µL mg^−1^	412 mAh g^−1^ (1 C, 1000 cycles)	0.041%	[72]
TiC@C‐TiO_2_	Cathode	2.3 mg cm^−2^ (74.2%)	10 µL mg^−1^	603 mAh g^−1^ (0.5 C, 160 cycles)	0.148%	[73]
ANDC/TiO_2−_ *_x_*	Cathode	1.8 mg cm^−2^ (75%)	NA	995 mAh g^−1^ (0.5 C, 500 cycles)	0.042%	[74]
TiO_2_‐CNFs@void@TiN@C	Separator	1.5 mg cm^−2^ (68%)/50 µm	20 µL mg^−1^	676 mAh g^−1^ (1 C, 1000 cycles)	0.054%	[75]
OV‐TiO_2−_ *_x_* @NC	Cathode	1.6 mg cm^−2^ (78.3%)	15 µL mg^−1^	792 mAh g^−1^ (1 C, 2000 cycles)	0.013%	[18b]
CCC@TiO_2_‐TiN	Cathode	3.5 mg cm^−2^ (60%)	NA	821 mAh g^−1^ (0.5 C, 500 cycles)	0.071%	[76]
Co_3_O_4_‐TiO_2_‐HPs	Cathode	1.0 mg cm^−2^ (75%)	NA	416 mAh g^−1^ (10 C, 500 cycles)	0.07%	[77]
H‐TiO_2_/r‐GO‐1	Cathode	1.5 mg cm^−2^ (80%)	15 µL mg^−1^	656 mAh g^−1^ (1 C, 1000 cycles)	0.023%	[78]
MnO_2_@HCF	Cathode	3.5 mg cm^−2^ (80%)	NA	662 mAh g^−1^ (0.5 C, 300 cycles)	0.088%	[79]
PEDOT/MnO_2_	Cathode	NA (87%)	NA	545 mAh g^−1^ (0.5 C, 200 cycles)	0.20%	[80]
MnO_2_/GO/CNTs	Cathode	2.8 mg cm^−2^ (90%)	NA	963 mAh g^−1^ (0.2 C, 100 cycles)	0.239%	[81]
HCNF@*δ*‐MnO_2_	Separator	2.2 mg cm^−2^ (80%)/2 µm	NA	856 mAh g^−1^ (0.5 C, 200 cycles)	0.13%	[82]
p‐CNT@Void@MnO_2_	Cathode	0.65–1.06 mg cm^−2^ (64.9%)	NA	526 mAh g^−1^ (1 C, 100 cycles)	0.13%	[83]
NHCSs@MnO_2_	Cathode	1.9 mg cm^−2^ (69.5%)	10 µL mg^−1^	737 mAh g^−1^ (0.5 C, 1000 cycles)	0.041%	[84]
G/CNT@MnO_2_	Cathode	1.5–2 mg cm^−2^ (81.8%)	NA	591 mAh g^−1^ (1 C, 200 cycles)	0.39%	[85]
MnO_2_@d‐Ti_3_C_2_	Cathode	3.7 mg cm^−2^ (69.5%)	NA	474 mAh g^−1^ (1 C, 500 cycles)	0.052%	[86]
NMRC@MnO_2_	Cathode	1.8 mg cm^−2^ (72%)	NA	590 mAh g^−1^ (2 C, 1000 cycles)	0.045%	[87]
PANI‐MnO_2_	Cathode	1.5 mg cm^−2^ (66%)	15 µL mg^−1^	826 mAh g^−1^ (1 C, 500 cycles)	0.055%	[88]
MnO_2_@rGO	Cathode	4 mg cm^−2^ (70%)	4 µL mg^−1^	578 mAh g^−1^ (0.2 C, 100 cycles)	0.17%	[89]
3DIS@MnO_2_	Cathode	1.4 mg cm^−2^ (91.5%)	NA	409 mAh g^−1^ (10 C, 900 cycles)	0.059%	[90]
YSC@Fe_3_O_4_	Cathode	5.5 mg cm^−2^ (80%)	NA	854 mAh g^−1^ (0.1 C, 200 cycles)	0.11%	[91]
PG‐450‐Fe_3_O_4_	Separator	0.6 mg cm^−2^ (60%)/15 µm	NA	732 mAh g^−1^ (1 C, 500 cycles)	0.027%	[92]
Fe_3_O_4_‐NC@ACC	Cathode	4.7 mg cm^−2^ (67%)	NA	780 mAh g^−1^ (0.2 C, 1000 cycles)	0.03%	[93]
Fe_3_O_4_/CNSs	Separator	1.5 mg cm^−2^ (70%)/8.5 µm	12 µL mg^−1^	610 mAh g^−1^ (1 C, 1000 cycles)	0.027%	[94]
Fe_3_O_4_‐PNCT‐1	Cathode	1.5 mg cm^−2^ (70%)	NA	612 mAh g^−1^ (1 C, 1000 cycles)	0.03%	[95]
3DOMPPy@ZnO	Cathode	NA (60.7%)	NA	795 mAh g^−1^ (0.1 C, 300 cycles)	0.06%	[96]
1D ZnO/2D G	Separator	1.1 mg cm^−2^ (70%)/71.2 µm	25 µL mg^−1^	765 mAh g^−1^ (2 C, 300 cycles)	0.12%	[97]
rGO@ZnO QDs	Cathode	1 mg cm^−2^ (70%)	NA	674 mAh g^−1^ (1 C, 400 cycles)	0.067%	[98]
N‐Co_3_O_4_@N‐C/rGO	Cathode	5.89 mg cm^−2^ (75%)	NA	568 mAh g^−1^ (0.2 C, 500 cycles)	0.062%	[99]
Co_3_O_4_/ACNT	Cathode	1.1 mg cm^−2^ 1.2 (58.73%)	NA	694 mAh g^−1^ (0.2 C, 550 cycles)	0.056%	[100]
RCE‐Co_3_O_4_@G	Cathode	0.8 mg cm^−2^ (71.63%)	NA	727 mAh g^−1^ (0.2 C, 350 cycles)	0.026%	[101]
Co_3_O_4_/C	Cathode	1.4 mg cm^−2^ (70%)	20 µL mg^−1^	520 mAh g^−1^ (1 C, 500 cycles)	0.083%	[102]
MoO_3_/MoO_2_‐CP	Cathode	NA (65.5%)	NA	828 mAh g^−1^ (0.5 C, 500 cycles)	0.016%	[103]
MoO_2_@CNT	Separator	1.7 mg cm^−2^ (75%)/15 µm	NA	540 mAh g^−1^ (1 C, 700 cycles)	0.066%	[104]
CeO_2_/MMNC	Cathode	3.4 mg cm^−2^ (70%)	NA	611 mAh g^−1^ (0.5 C, 200 cycles)	0.043%	[6a]
CeO_2_/CNF	Cathode	8.6 mg cm^−2^ (70.2%)	NA	897 mAh g^−1^ (0.1 C, 30 cycles)	0.76%	[105]
CNTs/SnO_2_ QDs	Cathode	2 mg cm^−2^ (70.3%)	NA	550 mAh g^−1^ (0.1 C, 700 cycles)	0.092%	[106]
T‐PPy@SnO_2_	Cathode	2 mg cm^−2^ (64.7%)	NA	542 mAh g^−1^ (1 C, 500 cycles)	0.05%	[107]
ALD‐V_2_O_5_@3DNG	Cathode	3.3 mg cm^−2^ (80%)	NA	542 mAh g^−1^ (2 C, 350 cycles)	0.053%	[108]
V_2_O_5_ NWs/GNS	Separator	1.5 mg cm^−2^ (70%)/40 µm	NA	326 mAh g^−1^ (2 C, 1000 cycles)	0.061%	[109]
HCS@Ti_4_O_7_	Cathode	NA (70%)	NA	609 mAh g^−1^ (0.5 C, 800 cycles)	0.06%	[110]
CF@CNTs mg^−1^O	Cathode	1.2 mg cm^−2^ (49%)	NA	390 mAh g^−1^ (2 C, 800 cycles)	0.06%	[111]
NiO‐CNT	Cathode	2.1 mg cm^−2^ (64.8%)	16 µL mg^−1^	609 mAh g^−1^ (0.1 C, 160 cycles)	0.216%	[112]
MCM/Nb_2_O_5_	Cathode	1.5 mg cm^−2^ (60%)	NA	650 mAh g^−1^ (2 C, 500 cycles)	0.092%	[113]
KB/Fe_2_O_3−_ *_x_*	Cathode	12.73 mg cm^−2^ (70%)	10 µL mg^−1^	612 mAh g^−1^ (0.05 C, 60 cycles)	0.21%	[114]
10La_2_O_3_‐NMC	Cathode	NA (60%)	NA	475 mAh g^−1^ (5 C, 100 cycles)	0.21%	[115]
G/G‐V_2_O_3_	Cathode	1.4–1.6 mg cm^−2^ (78.3%)	10 µL mg^−1^	540 mAh g^−1^ (2 C, 1000 cycles)	0.046%	[116]

NA: not available

### 3.1 Titanium Oxides

**Figure 7:**
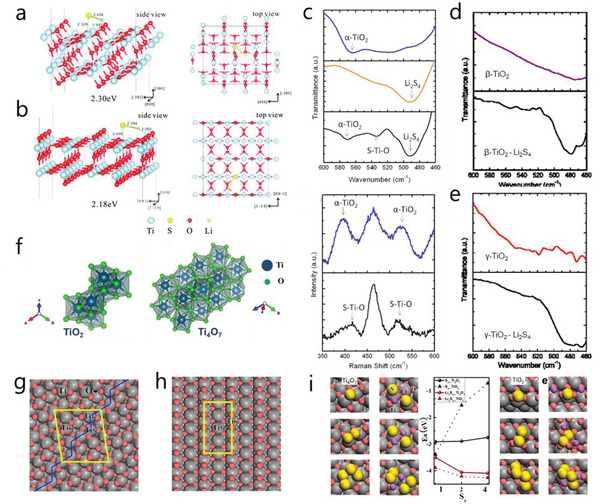
Adsorption configuration of LiS· on a) anatase (101) and b) rutile (110) TiO_2_. Reproduced with permission.^[^ ^118^ ^]^ Copyright 2016, Royal Society of Chemistry. c) FTIR and Raman spectra of *α*‐TiO_2_ and *α*‐TiO_2_/Li_2_S_4_ composite. FTIR spectra of d) *β*‐TiO_2_ and *β*‐TiO_2_/Li_2_S_4_ and e) *γ*‐TiO_2_ and *γ*‐TiO_2_/Li_2_S_4_. Reproduced with permission.^[^ ^58^ ^]^ Copyright 2012, American Chemical Society. f) Crystal structure of TiO_2_ and Ti_4_O_7_. Reproduced with permission.^[^ ^124^ ^]^ Copyright 2022, Wiley‐VCH. Schematics of g) Ti_4_O_7_ (1–20) and h) TiO_2_ (110) surfaces. i) DFT analysis of the adsorption to S species of Ti_4_O_7_ and TiO_2_, respectively. Reproduced with permission.^[^ ^122^ ^]^ Copyright 2014, American Chemical Society.

TiO_2_ is the natural oxide of titanium with four types of crystallographic forms including anatase (*α*‐TiO_2_), rutile (*β*‐TiO_2_), brookite (*γ*‐TiO_2_) and bronze (B‐TiO_2_), where the bronze is the least stable.^[^ ^117^ ^]^ DFT calculations have shown that the binding energies between *α*‐ and *β*‐TiO_2_ to LiS· (represent LiPSs) is 2.30 and 2.18 eV, respectively (**Figure** **7**a,b).^[^ ^118^ ^]^ In the *α*‐TiO_2_/Li_2_S_4_ composite, the S—Ti—O signal and Raman peak shifts suggest bond formation between *α*‐TiO_2_ and Li_2_S_4_ (Figure 7c). However, the signal could not be detected in either the *β*‐ or *γ*‐TiO_2_/Li_2_S_4_ composite (Figure 7d,e), implying weaker bindings with Li_2_S_4_ than *α*‐TiO_2_.^[^ ^58^ ^]^ Similarly, the adsorption behavior of B‐TiO_2_ was also evaluated by DFT calculation, showing a weaker binding with Li_2_S_8_ (1.63 eV) than that of *α*‐TiO_2_ (1.83 eV). However, when CNT@B‐TiO_2_ and CNTs@*α*‐TiO_2_ were designed as separator interlayer, respectively, CNTs@B‐TiO_2_ delivered a superior performance with higher capacity and more stable cycling than CNTs@*α*‐TiO_2_. Such result was attributed to the diverse diffusion properties. DFT analysis demonstrated a smaller Li^+^ diffusion barrier of B‐TiO_2_ (0.58 eV) than *α*‐TiO_2_ (0.71 eV), highlighting the importance of diffusion properties of MOs in the typical adsorption–diffusion–conversion process.^[^ ^119^ ^]^

Despite effective entrapment of LiPSs, the low conductivity of TiO_2_ renders quite undesirable electrochemical performance. In contrast, titanium based oxide Ti_4_O_7_ possesses high conductivity even showing metallic properties (Figure 7f).^[^ ^120^ ^]^ When Ti_4_O_7_ is directly employed as sulfur host, the Ti_4_O_7_/S provides a reversible discharge capacity of 1070 mAh g^−1^ at 0.2 C, and reversible cyclic stability of over 500 cycles.^[^ ^121^ ^]^ Besides conductivity, Ti_4_O_7_ also exhibits a rare sulfiphilic surface different from most MOs with a lithophilic surface, which enables Ti_4_O_7_ to form S bonds with LiPSs rather than Li bonds. To understand the surface properties, comparison is made between TiO_2_ (rutile as example) and Ti_4_O_7_. As shown in Figure 7g, three different Ti atoms of Ti_4c_, Ti_5c_ and Ti_6c_ represent the coordination number of 4, 5 and 6, respectively, where Ti_4c_ and Ti_5c_ is unsaturated. TiO_2_ contains Ti_5c_ and Ti_6c_ in a ratio of 1:1, suggesting the unsaturated Ti fraction is 50%. Whereas in Ti_4_O_7_ (Figure 7h), the unsaturated Ti ratio is 62.5%, with low‐coordinated Ti_4c_ and Ti_5c_ arranged in the step sites highlighted as a blue line. DFT calculations suggest that the moderate S bond dominates the interaction with LiPSs rather than the Li bond on the Ti_4_O_7_ surface (Figure 7i).^[^ ^122^ ^]^

The metallic Ti_4_O_7_ can afford a sulfiphilic surface, which is promising as a sulfur host in Li—S batteries. However, the fabrication of Ti_4_O_7_ was complex, generally requiring harsh conditions such as high temperatures of around 1000 °C and a pure reduction atmosphere.^[^ ^14^ ^]^ Although relatively cost‐effective mild methods were proposed recently,^[^ ^123^ ^]^ it is still a challenge to construct a nanostructured and flexible Ti_4_O_7_ conductive framework.

### 3.2 Manganese Oxides

**Figure 8:**
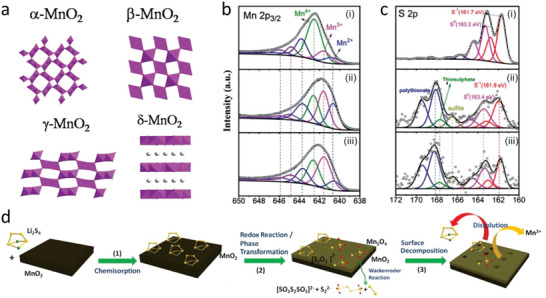
a) The structure of *α*, *β*, *γ*, and *δ*‐MnO_2_. Reproduced with permission.^[^ ^125^ ^]^ Copyright 2015, Royal Society of Chemistry. b) Mn 2*p*_2/3_ XPS spectrum of i) *γ*‐MnO_2_, ii) *γ*‐MnO_2_‐Li_2_S_4_ after 0.5 h, and iii) *γ*‐MnO_2_‐Li_2_S_4_ after 5 days. c) S 2*p* spectrum of i) Li_2_S_4_, ii) *γ*‐MnO_2_‐Li_2_S_4_ after 0.5 h, and iii) *γ*‐MnO_2_‐Li_2_S_4_ after 5 days. d) Schematic illustration of the interaction between *γ*‐MnO_2_ and Li_2_S_4_, which concomitant with the surface decomposition of Mn_3_O_4_. Reproduced with permission.^[^ ^126^ ^]^ Copyright 2017, Wiley‐VCH.

MnO_2_ is the most common manganese based oxide, which occurs naturally as blackish or brown mineral pyrolusite. Similarly, MnO_2_ also has four common polymorphs (*α*, *β*, *γ*, and *δ*‐MnO_2_, **Figure** **8**a).^[^ ^125^ ^]^ It is worth noting that the surface of MnO_2_ is always nonstoichiometric with oxygen deficiency.^[^ ^14^ ^]^ Benefited with its unique characteristics, MnO_2_ is effective in the Li—S catalytic process and was extensively investigated and utilized in Li—S batteries. For example, *δ*‐MnO_2_ nanosheets were initially applied as sulfur host to investigate the adsorption to LiPSs.^[^ ^48^ ^]^ As detailed before, MnO_2_ can act as a prototype to react with initially formed LiPSs to generate the surface‐bound intermediate thiosulfate, which serves as a redox mediator to further catenate and react with LiPSs via disproportionation. It was proposed that only metal oxides with a suitable redox potential window can trigger the thiosulfate formation.^[^ ^51a^ ^]^ Likewise, this so called “Wackenroder reaction” in *δ*‐MnO_2_ was also found in *γ*‐MnO_2_. It was found that the surface phase transformation from MnO_2_ to Mn_3_O_4_ was accompanied by the redox reaction between LiPSs and prototype *γ*‐MnO_2_ host.^[^ ^126^ ^]^ As shown in Figure 8b, compared with pristine MnO_2_, after being treated with LiPSs, MnO_2_ was partially converted into lower valence species with significantly increased Mn^3+^ and Mn^2+^ peaks, which is accordant with the generation of Mn_3_O_4_ detected by XRD. Meanwhile, in the S 2*p* spectrum (Figure 8c), Li_2_S_4_ was oxidized into thiosulfate/polythionate species. Thus, the initial MnO_2_ partially reacts with Li_2_S_4_ into thiosulfate/polythionate species and Mn_3_O_4_, which further facilitates LiPSs conversion. Notably, the decreased intensity of Mn^2+^ (Figure 8b) suggests the dissolution of Mn^2+^ in DME, which would result in a slight capacity degradation. The whole process for the proposed mechanism is depicted in Figure 8d. *α* or *β*‐MnO_2_ have also been investigated in Li—S batteries.^[^ ^127^ ^]^ Although numerous works were established for the MnO_2_, however, the interactions and catalytic effect between different crystal phases (*α*, *β*, *γ*, and *δ*) and LiPSs are yet to be resolved.

### 3.3 Iron and Cobalt Oxides

**Figure 9:**
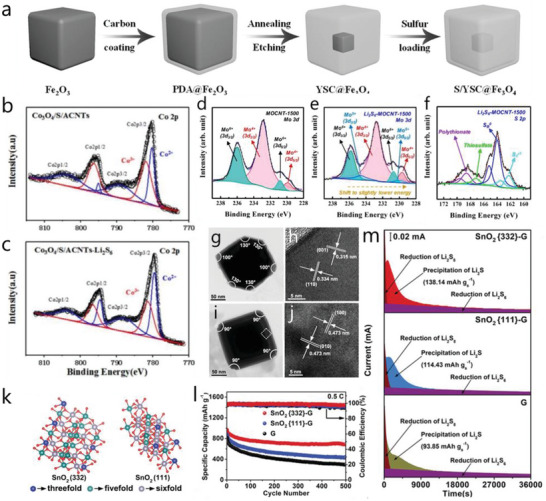
a) Schematic diagram of synthesis of S/YSC@Fe_3_O_4_ composite. Reproduced with permission,^[^ ^91^ ^]^ Copyright 2017, Wiley‐VCH. XPS spectra of Co 2*p* in b) Co_3_O_4_/S/ACNT and c) Co_3_O_4_/S/ACNT‐Li_2_S_4_ composites. Reproduced with permission.^[^ ^100^ ^]^ Copyright 2019, American Chemical Society. XPS spectra of Mo 3*d* in d) MOCNT‐1500 and e) Li_2_S_6_‐MOCNT‐1500 composite. f) XPS spectra of S 2p in Li_2_S_6_‐MOCNT‐1500 composite. Reproduced with permission.^[^ ^104^ ^]^ Copyright 2020, American Chemical Society. g,i) TEM images of SnO_2_ (332) and (111) facets and h,j) the corresponding HRTEM images of the selected region. k) Schematic model of SnO_2_ (332) and (111) facets. l) Cyclic performances of SnO_2_‐G (332) and SnO_2_‐G (111) cells at 0.5 C. m) Potentiostatic precipitation of SnO_2_ (332)‐G, SnO_2_ (111)‐G, and G electrode. Reproduced with permission.^[^ ^133^ ^]^ Copyright 2021, Wiley‐VCH.

Iron‐based oxides, for example wüstite (FeO), hematite (*α*‐Fe_2_O_3_), maghemite (*γ*‐Fe_2_O_3_) and magnetite (Fe_3_O_4_) with abundant reserves and low costs, possess an excellent polar adsorption and catalytic effect to LiPSs.^[^ ^128^ ^]^ Among them, Fe_3_O_4_, the most widely studied iron oxide, possesses a relatively high electronic conductivity and prominent anchoring ability. Due to the superior advantages of both high polarity and conductivity, Fe_3_O_4_ based conductive compounds are expected to realize high performance in Li—S batteries. For example, a yolk‐shelled carbon@Fe_3_O_4_ nanobox was designed as sulfur host (**Figure** **9**a). With the chemical interaction in the Fe_3_O_4_ core and physical blocking in the carbon shell, this synergistic effect enabled a high sulfur loading and sulfur content (5.5 mg cm^−2^ and 80 wt%), achieving a high area capacity of 6.97 mAh cm^−2^ at 0.1 C and decent stability over 200 cycles.^[^ ^91^ ^]^ Recently, as metal‐organic frameworks (MOFs) have attracted public attention because of their controllable porous structures and porosities, a MOF‐derived carbon encapsulated Fe_3_O_4_ (Fe_3_O_4_@C) was proposed as sulfur host to mediate polysulfide redox reaction. Benefitting from the strong adsorption of metal oxides and hierarchical porous structures inheriting from MOFs, an excellent performance was achieved.^[^ ^129^ ^]^ In addition, it is proposed to be effective to couple a carbon substrate with ultra‐small Fe_3_O_4_ nanoparticles where Fe_3_O_4_ NPs act as an efficient LiPSs trapping and active center, promoting the utilization of sulfur materials,^[^ ^93^, ^94^, ^128^, ^129^, ^130^ ^]^ which gained much attention from researchers.

Similar to Fe_3_O_4_, Co_3_O_4_ also gained attention due to higher conductivity than most metal oxides and similar non‐stoichiometric characteristics with two different valance pair states of metal. Recently, researchers found that the valance pair state of Co^2+^ and Co^3+^in Co_3_O_4_ can serve as redox pair that can affect the redox of LiPSs in Li—S chemistry. Figures 9b and 9c show the XPS spectra of Co 2*p* in Co_3_O_4_ and Co_3_O_4_‐Li_2_S_4_, where the peaks of Co_3_O_4_ can be divided into two valance states of Co^2+^ and Co^3+^. After contacting with Li_2_S_6_, the intensity of the Co^3+^ peak decreased and Co^2+^ increased, which results in the appearance of thiosulfate and polythionate, thus demonstrating the electron transfer from Li_2_S_6_ to Co_3_O_4_.^[^ ^100^ ^]^ Such phenomenon is similar to that of MnO_2_, where MnO_2_ also displays multiple valance states at the surface with a main valance of Mn^4+^ and other valances of Mn^3+^ and Mn^2+^.^[^ ^126^ ^]^ Due to the superior properties of Co_3_O_4_, decent performances could be realized. Park et al. designed a multidimensional architecture with N‐doped graphene and Co_3_O_4_ nanoparticles encapsulated CNT branches to combine the strong affinity and enhanced kinetics of Co_3_O_4_ and confined porous structure, realizing a capacity of 5.62 mAh cm^−2^ at the high loading of 6.5 mg cm^−2^.^[^ ^131^ ^]^

### 3.4 Other Metal Oxides

MoO_2_ has an oxidized surface where Mo^6+^ and Mo^4+^ coexist, which give rise to high LiPSs conversion kinetics. Figure 9d,e shows the Mo 3*d* in XPS of MoO_2_ and MoO_2_‐Li_2_S_6_ based composite, respectively. Compared with MoO_2_, after interaction with Li_2_S_6_, a slight decrease of the Mo^6+^ peak and a newly appearing Mo^5+^ state were found, which indicates a surface redox reaction between MoO_2_ and Li_2_S_6_. Besides, the XPS in S 2*p* shows the formation of thiosulfate/polythionate in Li_2_S_6_ after contacting with MoO_2_ (Figure 9f), which facilitates the LiPSs adsorption and redox conversion.^[^ ^104^ ^]^ Lanthanide oxides stand out as efficient catalysts in electrocatalysis due to the moderate band gap as well as multiple valance states,^[^ ^132^ ^]^ where CeO_2_ is prominent as LiPSs catalyst. Generally, on CeO_2_ surface, except Ce^4+^ valance, Ce^3+^ also exist associated with oxygen vacancies.^[^ ^132b^ ^]^ The high catalytic ability enables a decent applicable Li—S battery performance. For example, a CeO_2_ decorated carbon nanofiber as sulfur host (S@CeO_2_@CNF) was designed and achieved a high initial capacity of 8.4 mAh cm^−2^ at a high sulfur loading of 8.6 mg cm^−2^ and retained more than 30 cycles.^[^ ^105^ ^]^

SnO_2_ was considered as an efficient catalyst in Li—S chemistry. However, it was reported that the catalytic activity of SnO_2_ is greatly influenced by different crystal facets. As shown in Figure 9g–k, the nano‐octahedra SnO_2_ with two diverse crystal facets of [332] and [111] were typically synthesized and anchored on reduced graphene oxide (G). Compared with SnO_2_ [111]‐G and G electrode, SnO_2_ [332]‐G exhibited a more superior cyclic performance (Figure 9l). Moreover, in potentiostatic precipitation tests of Li_2_S, as shown in Figure 9m, the higher precipitation capacity of SnO_2_ [332]‐G reveals a lower nucleation barrier of Li_2_S, confirming the improved catalytic property. The improved catalytic activity and cyclic performance is attributed to the more abundant unsaturated‐coordinated Sn sites on the [332] plane. As a results, with a higher catalytic activity of [332], the SnO_2_ [332]‐G cell achieved the area capacity of 6.93 mAh cm^−2^ over 100 cycles with a high sulfur loading of 8.12 mg cm^2^.^[^ ^133^ ^]^

Except single metal oxides, the synergism of bimetallic oxides was also explored in terms of LiPSs engineering. A Fe and V coordinated bimetallic oxide FeVO_4_ nanocatalyst with 3D ordered structure was used to modify the separator for achieving the restraining of LiPSs diffusion and boosting the conversion kinetics of sulfur species. It was demonstrated that the FeVO_4_ can effectively enhance the anchoring and catalytic activity toward LiPSs compared with single metal oxides of Fe_2_O_3_ and V_2_O_5_. In Li—S pouch cells, the FeVO_4_/CNT modified separator delivered an initial capacity of 6.5 mAh cm^−2^ at 0.2 C under a high sulfur loading of 6.1 mg cm^−2^, lean electrolyte with E/S ratio of 5 µL mg^−1^ and N/P ratio of 2, achieving an energy density up to 341 Wh kg^−1^.^[^ ^134^ ^]^

The above discussion provides examples using various MOs catalysts employed as sulfur host in cathodes or separator interlayers of Li—S batteries. In principle, to achieve a high sulfur loading and energy density performance, it is necessary to decrease the amount of non‐active material such as MOs or conductive substrate. Thus, developing effective MOs with high adsorption–catalytic activity is essential and considered to be the main topic of research. However, it is hard to make intuitive comparison and screen the most effective MOs electrocatalyst, because different bulk phases and crystal facets may deliver different activities. And other features like shapeable, durability, or availability also matter.

Nevertheless, it is worth noting that multiple valances on the surface of MOs are favored for realizing significant adsorption and catalytic properties toward LiPSs, where the redox valance pair would enable the formation of thiosulfate/polythionate, thus facilitating the adsorption–catalysis process. This can be recognized as a dual chemical adsorption mode which combines both sulfur‐chain catenation and Li/S bond mechanism. A similar example can be found in the comparison between MXene and GO substrates. Both of which can provide —OH groups that can catenate sulfur‐chain into the thiosulfate/polythionate mediator. However, after consuming the available —OH, the exposed active Mo sites on MXene can further proceed into a S bond for second adsorption,^[^ ^50^ ^]^ whereas the second adsorption in GO is unavailable, leading to inferior LiPSs adsorption–catalysis process than MXene. The introducing of the dual chemical adsorption mode is effective for realizing high adsorption–catalysis property.

As is known to all, a high utilization of MOs catalyst is essential to improve the cell performances. There are two cases for MOs catalysts used in Li—S batteries. The first is MOs serving as sulfur hosts. In this case, the low conductivity and low surface area are the main limitations. The common strategy is to fabricate 3D structures to increase the surface area as well as confining sulfur species, and to improve the conductivity of MOs host by introducing a reduction process step in synthesis or adding carbonaceous additives.^[^ ^135^ ^]^ A typical example is the fabrication of 3D ordered macroporous Nb_2_O_5−_ *_x_* architecture combined with CNTs embedded in the framework to enhance the surface area and conductivity.^[^ ^136^ ^]^ After compositing with sulfur by a melting method, the assembled cells could achieve superior performances. Another case is MOs catalysts serving as additives, with carbonaceous materials as sulfur host.^[^ ^16^, ^137^ ^]^ MOs are used to decorate the surface of the carbon host, this case is much more common. However, due to the inherent low conductivity of MOs, the LiPSs on MOs will go through an “adsorption‐diffusion‐conversion” process, in which diffusion is an additional step with sluggish kinetics. Therefore, introducing fine and nanostructured MOs catalyst to shorten the diffusion path is an efficient way to improve the utilization of catalysts. Besides, changing the conductive properties to directly realize the nucleation of Li_2_S upon the MOs surface is also available.^[^ ^15a^ ^]^

No matter if MOs serve as sulfur host or additives, the nanostructured MOs is conduced to the performance for exposing more active sites and physically confining LiPSs. However, the low conductivity and limited catalytic activity still restrict further development and application. To enhance the intrinsic catalytic properties, as well as the side effect by the insulating nature, two main strategies with tuning the basic electron structures are proposed here: 1) constructing oxygen vacancies (OVs) that can improve the conductivity of MOs themselves, as well as enhancing the adsorption and catalytic activity; and 2) by combining another high conductive component to form heterostructures, the nucleation sites could be relocated resulting in a reduced diffusion barrier. In the meanwhile, the formed heterojunction shows a high adsorption–catalytic activity. These two strategies are considered to be effective and will be explained in detail in the following sections.

## 4 Oxygen Vacancies (OVs) Engineering

### 4.1 Basic Mechanisms of Oxygen Vacancies in Li‐S Chemistry

As discussed above, some MOs (MnO_2_, CeO_2_, etc.) containing OVs on the surface result in multiple valance states of the M cation, which play a determining role in the catalytic properties. Beyond that, creating rich OVs in bulk MOs is expected to alter the intrinsic electrical conductivity as well as the coordination state toward LiPSs. A typical example is the synthesis of a series of substoichiometric phases Ti*_n_* O_2_ *_n_*_−1_ from TiO_2_ called Magnéli phases, possessing an improved conductivity with respect to TiO_2_. One of its members, Ti_4_O_7_ even exhibits a metallic conductivity and shows a different sulfiphilic surface. Therefore, OVs play a key role in tailoring the catalytic properties, which can modulate intrinsic conductivity, coordination state toward LiPSs, and even influence the mechanism of adsorption–diffusion–catalysis.

**Figure 10:**
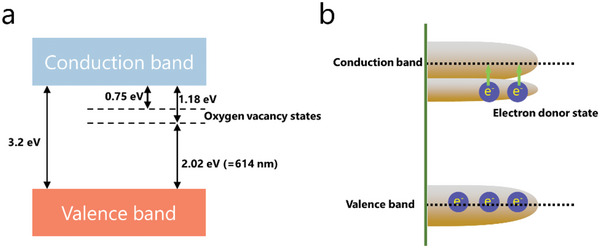
a) A proposed model of band structure of oxygen vacancy state in anatase‐TiO_2_. b) The overlapping of donor state and conduction band.

In order to figure out the role of OVs in the band structure, pioneering works have contributed to reveal the band changes in the vacancies of TMOs species.^[^ ^138^ ^]^ Generally, by removing an oxygen atom from the bulk lattice, the position previously occupied by O^2−^ in the regular lattice would be filled by excess “free” electrons to minimize the formation energy of the vacancy, leading to localized electrons in the vacancy state.^[^ ^138a^ ^]^ Nakamura and co‐workers used to proposed a band structure model to understand the localized electrons in anatase TiO_2_, where the energy level of the localized donor states of the oxygen vacancy is located 0.75–1.18 eV below the conduction band, as shown in **Figure** **10**a.^[^ ^138c^ ^]^ On the other hand, the OVs can also cause redistribution of electrons among adjacent metal atoms to form shallow donor states below the conduction band. For anatase it was demonstrated that the energy of these donor states would rise and even overlap with the conduction band at higher vacancy concentration (Figure 10b).^[^ ^139^ ^]^ As a result, the formation of these electron donor states will result in n‐doping, providing an explanation to the enhanced conductivity for rich‐OVs MOs. In *d*‐band theory, the electron donor states below the conduction band will also influence the *d* band center of the metal atoms that shift toward the Fermi level, which gives rise to a higher adsorption and catalytic activity to LiPSs.

### 4.2 Synthetic Strategies of Oxygen Vacancies

To construct OVs, thermal treatment of MOs under reducing atmosphere is a common strategy and has been proven to be an effective method. For example, by annealing TiO_2_ nanosheets in Ar/H_2_ atmosphere at various temperatures, different vacancy concentrations in TiO_2_ could be generated.^[^ ^140^ ^]^ It is worth noting that the H_2_ can also be replaced by other reducing atmospheres like CO, NH_3_, CH_4_, and so forth.^[^ ^139^, ^141^ ^]^ Nevertheless, it is quite challenging to infer how the reduction atmosphere or annealing temperature influences the extent of OVs. In other specific cases, the reducing atmosphere is not even necessary. The oxygen deficient atmosphere (or vacuum) allows the oxygen atom to escape from the MOs lattice, leading to the generation of OVs. For example, by annealing hexagonal WO_3_ nanorods in a vacuum environment, monoclinic WO_3−_ *_x_* can be obtained by phase transformation.^[^ ^142^ ^]^

Except annealing, OVs can also be introduced by solution methods, using reduction reagents such as NaBH_4_, KBH_4_, N_2_H_4_, etc. For example, TiO_2−_ *_x_* could be directly obtained by adding TiO_2_ nanosheets into a NaBH_4_ solution at room temperature.^[^ ^143^ ^]^ Compared with the above complicated annealing strategy, the solution method is much more facile. In addition, electrochemical reduction is an alternative green strategy to produce OVs with a scalable and faster preparation. For instance, the oxygen‐deficient TiO_2−_ *_x_*, WO_3−_ *_x_*, BiVO_4−_ *_x_* and ZnO_1−_ *_x_* can be fabricated by applying specific potentials to TiO_2_, WO_3_. BiVO_4_ and ZnO in aqueous electrolyte solution.^[^ ^144^ ^]^

Apart from post‐treatment of MO, OVs can also be created during the synthesis of MO, forming MO*_x_*. By inhibiting nucleation and crystalline growth at the atomic level, defects in the form of vacancies can be generated. Such inhibition processes include solvothermal,^[^ ^145^ ^]^ ultrasonication,^[^ ^146^ ^]^ ball milling,^[^ ^147^ ^]^ and others.^[^ ^148^ ^]^ Thus, these MO*_x_* were synthesized by controlling the conversion condition in the process of precursors' transformation.

### 4.3 Recent Progresses of Oxygen Vacancies in Li‐S Chemistry

**Figure 11:**
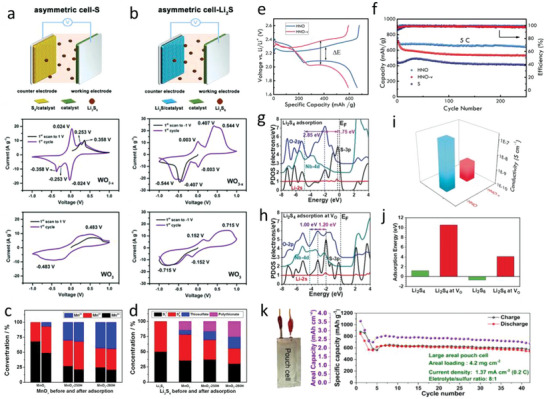
Cyclic voltammograms of asymmetric cell a) ‐S and b) ‐Li_2_S with WO_3−_ *_x_* and WO_3_ in Li_2_S_6_ electrolyte. Reproduced with permission.^[^ ^149^ ^]^ Copyright 2018, Wiley‐VCH. Comparison of concentration of c) Mn valance states in MnO*_x_* and d) S valance states in Li_2_S_4_ before and after adsorption. Reproduced with permission.^[^ ^151^ ^]^ Copyright 2019, Elsevier. Comparison of e) charge/discharge profile and f) cycling performance of HNO and HNO‐v cathode. g,h) The projected density of state (PDOSs) of Li_2_S_6_ on pristine and OVs‐HNb_3_O_8_. i) Electric conductivity and j) adsorption energy to Li_2_S_6_ of pristine and OVs‐HNb_3_O_8_. Reproduced with permission.^[^ ^152^ ^]^ Copyright 2019, Wiley‐VCH. k) The cycling performance of AOV‐Nb_2_O_5−_ *_x_* @HHPC@S cathode in pouch cell. Reproduced with permission.^[^ ^18c^ ^]^ Copyright 2021, Elsevier.

Up to now, oxygen vacancies in MOs are extensively exploited as active sites for anchoring and catalyzing the redox reactions in Li—S batteries. Since it was first utilized in Li—S chemistry to suppress the “shuttle effect” and improve the utilization of sulfur through enhancing the adsorption and catalytic activity in 2017,^[^ ^143^ ^]^ numerous works were published to demonstrate the effectivity for constructing OVs on MOs in Li—S chemistry. For example, Lee et al. studied the function of OVs in WO_3−_ *_x_* nanoplatelets by designing asymmetric cells. As shown in **Figures** **11**a,b, in WO_3−_ *_x_* and WO_3_ based asymmetric cells, the voltammogram of WO_3−_ *_x_* based electrodes show higher redox reversibility in both S and Li_2_S cells, confirming the bidirectional catalytic properties of OVs in Li—S chemistry.^[^ ^149^ ^]^ First principles DFT studies demonstrated that the formation of OVs in anatase TiO_2_ enhances the binding energy of polysulfides as well as the electronic conductivity.^[^ ^150^ ^]^ To further investigate the behaviors of OVs in the thiosulfate/polythionate formation process, MnO_2_ incorporating OVs was synthesized by heating MnO_2_ hollow nanospheres under reductive atmosphere.^[^ ^151^ ^]^ As shown in Figure 11c,d, where OVs‐rich MnO_2_ present multiple valence states of Mn^4+^, Mn^3+^ and Mn^2+^ on the surface, MnO_2_ only shows Mn^4+^ and Mn^3+^. After adsorption of Li_2_S_4_, similar redox behaviors were found where the contribution from Mn^4+^ sites decreases and the one of Mn^3+^ and Mn^2+^ sites increases. The higher thiosulfate/polythionate content in the case of OV‐rich MOs indicates accelerated formation of the S_2_O_3_^2−^ mediator, assisting a stronger adsorption of polysulfides as well as their dissociation, corroborating the high catalytic effect of OVs.

However, manufacturing oxygen vacancies is not always beneficial for adsorption and catalytic conversion. An exception was also reported for leading to inferior Li—S performance. As shown Figure 11e,f, after constructing fully oxidized HNb_3_O_8_ (HNO) and OVs rich HNb_3_O_8_ (HNO‐v) based sulfur electrodes, it can be seen that HNO‐v presents a larger polarization and poorer cyclic performance than HNO. Further electronic structure analysis (Figure 11g,h) provided an explanation that the overlap of O 2*p*_*π*_ and S 3*p*_*π*_ orbitals in HNO‐v induces a strong repulsion between the lone pair of electrons, deteriorating the adsorption–catalytic process. As a result, the OVs rich HNO‐v exhibits a decreased electric conductivity as well as a weakened adsorption to LiPSs (Figures 11i,j).^[^ ^152^ ^]^ Thus, care should be taken when designing new catalysts using the oxygen vacancy engineering strategy, where the electric conductivity and adsorption are the focusing issues.

By fabricating multifunctional hosts with OVs‐rich MOs and structured carbonaceous material, excellent electrochemical performances could be achieved in Li—S batteries. In a recent work, a kind of double shell nanotubes of titanium oxide with OVs in nitrogen‐doped carbon host (OVs‐TiO_2−_ *_x_* @NC) was prepared, which enabled a super high cycling stability of merely 0.0123% capacity fade per cycle within 3000 cycles at 5 C. When loading an impressively high amount of sulfur (9.5 mg cm^−2^), a high area capacity of 8.01 mAh cm^−2^ was achieved with a low E/S ratio of 5 µL mg^−1^.^[^ ^18b^ ^]^ Such superior electrochemical performances can be attributed to the rational integration of physical spatial confinement and high adsorption–catalytic effect of OVs‐TiO_2−_ *_x_*. Similarly, a multifunctional OVs‐niobium oxide electrocatalyst combined with hierarchical porous nanocarbon (AOV‐Nb_2_O_5−_ *_x_* @HHPC) was also fabricated to assess the electrochemical behavior in large area Li—S pouch cells. As shown in Figure 11k, the pouch cell achieved an initial area capacity of 3.54 mAh cm^−2^ and stabilized for more than 40 cycles with sulfur loading of 4.2 mg cm^−2^ at lean electrolyte, which provides a promising application for the future.^[^ ^18c^ ^]^ A similar pouch cell performance was also demonstrated in the Nb doped OVs‐TiO_2−_ *_x_* catalytic sulfur host, the fabricated S‐NC@Nb‐TiO_2−_ *_x_* electrode realized 809 mAh g^−1^ of capacity under 3.5 mg cm^−2^ of sulfur loading and 9.5 µL mg^−1^ of E/S ratio.^[^ ^59b^ ^]^ Therefore, we note that OVs play important roles in improving the adsorption–catalytic properties thus making it possible to achieve high performance Li—S batteries.

## 5 Heterostructure Engineering

### 5.1 Basic Heterostructure Strategies in Li‐S Batteries

Heterostructures which consist of two components or blocks forming a heterointerface through physical (mainly van der Waals [vdW] force) or chemical bonds, are an alternative promising strategy to address the insulating issue of MOs. It should be clarified that the heterostructure differs from a general composite material that also consists of two different components. The design principle of such a composite material is simply combining the complementary advantages of each component. For instance, MOs with strong LiPSs adsorption are usually combined with a conductive substrate to form a hybrid composite realizing both high adsorption and electron transport properties, while the focus of heterostructures is quite different, mainly on the effect of heterointerface.

**Figure 12:**
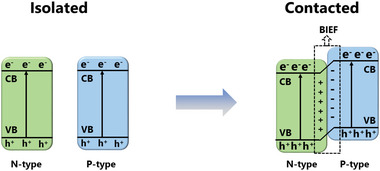
The diagram of formed BIEF after two types of building blocks contact with each other to form a heterojunction.

Due to the different properties (e.g., band structure, carrier concentration, semiconductor type, Fermi level, alignment style) of building blocks, once brought into contact, the band alignments at the interface proceeds till the Fermi level reaches equilibrium. The band structure of building blocks would be bent resulting in a built‐in electric field (BIEF) at the heterointerface (**Figure** **12**). The heterojunction between two components forming the BIEF are revealed to specifically facilitate adsorption and reduction/oxidation reactions of redox species.^[^ ^153^ ^]^ For example, Sun et al. designed a kind of Mott–Schottky heterojunction and investigated the potential catalytic activity in Li—S chemistry. They found that the BIFE with negatively and positively charged interfaces can strongly adsorb Li^+^ and polysulfide anions of LiPSs, respectively, thus lowing the energy barriers of sulfur redox reactions and accelerating catalytic conversion.^[^ ^154^ ^]^ The resulting heterostructure provides both the sum of the contribution of the individual components and the heteroconjunction at their interface with completely improved properties, realizing a performance superior to the sum of its part (“1+1>2”).

### 5.2 Synthesis Strategies of MO Based Heterostructures

**Figure 13:**
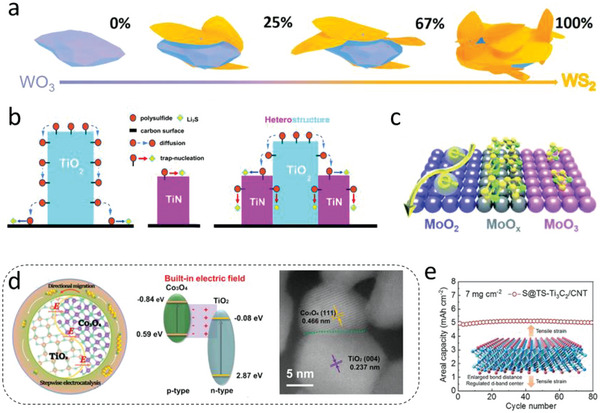
a) The illustration of component change with different sulfurization degree. Reproduced with permission.^[^ ^155^ ^]^ Copyright 2020, Wiley‐VCH. b) Schematic of principle of LiPSs conversion process on TiO_2_–TiN heterostructure. Reproduced with permission.^[^ ^15b^ ^]^ Copyright 2017, Royal Society of Chemistry. c) Schematic of MoO_3_/MoO_2_ heterostructure with a MoO*_x_* transition state. Reproduced with permission.^[^ ^103^ ^]^ Copyright 2020, Royal Society of Chemistry. d) Schematic diagram of Co_3_O_4_ and TiO_2_ heterostructure with BIEF. Reproduced with permission.^[^ ^77^ ^]^ Copyright 2021, Wiley‐VCH. e) Illustration and the high loading performance of S@TS‐Ti_3_C_2_/CNT. Reproduced with permission.^[^ ^160^ ^]^ Copyright 2021, Wiley‐VCH.

Different from physically mixing of two components, heterostructures are formed with conjunction between the two blocks guaranteeing the close contact. To realize this, partial sulfurization, reduction, or nitrification has attracted much attention. To take an example, as shown in **Figure** **13**a, a type of WS_2_–WO_3_ heterostructure was reported by in situ sulfurization of WO_3_. By controlling the weight ratio and annealing temperature, an adjustable constituent ratio of WS_2_–WO_3_ heterostructure could be obtained.^[^ ^155^ ^]^ Another typical example is the synthesis of the MOs‐MXene heterostructure by partial oxidation of MXene. When treating Ti_3_C_2_T*_x_* (T is a terminal as functional groups) by a hydrothermal process, TiO_2_ nanoparticles were nucleated on the surface of Ti_3_C_2_T*_x_* to form a TiO_2_‐Ti_3_C_2_T*_x_* heterostructure.^[^ ^156^ ^]^ Similarly, a Nb_2_O_5_‐NbC_3_T*_x_* heterostructure was also reported with Nb_2_O_5_ nanoparticles uniformly distributed on the surface of NbC_3_T*_x_* by heating Nb_4_C_3_T*_x_* in CO_2_ flow.^[^ ^157^ ^]^ In addition, other methods like atomic layer deposition (ALD), chemical vapor deposition (CVD), and so on can also produce a heterostructure material with one component onto a target substrate.^[^ ^158^ ^]^

Aside from above strategies, heterostructure material can also be synthesized through a one step process such as annealing or hydrothermal treatment. And the resulting product can be controlled by the ratio of reactants or the reaction conditions. For example, a TiO_2_–TiN heterostructure was synthesized by the reaction between urea and TiCl_4_ annealed under N_2_ atmosphere, where the resulting product can be determined by the molar ratio of urea/TiCl_4_. When the ratio was between 2:1 and 10:1, the TiO_2_–TiN heterostructure can be obtained.^[^ ^15b^ ^]^ Another example is the MoO_2_/*α*‐MoC heterostructure constructed by the thermal decomposition of Mo_3_O_10_(C_6_H_8_N)·2H_2_O in Ar atmosphere where the resulting product is temperature dependent. Annealing temperatures of 600 and 700 °C would result in pure MoO_2_ and *α*‐MoC, respectively. Thus, MoO_2_/*α*‐MoC heterostructures can be obtained when the temperature was set at 650 °C.^[^ ^159^ ^]^

### 5.3 Recent Progresses of MO Based Heterostructures in Li‐S Chemistry

In a typical adsorption–diffusion–conversion process of LiPSs in MO based electrocatalysts, LiPSs must diffuse from nonconductive metal oxide to the conductive carbon surface to realize further conversion. This process always shows slow reaction kinetics. Aiming to solve this problem, the concept of heterostructure was initially introduced by Yang's group in Li—S chemistry to smooth such diffusion‐conversion process.^[^ ^15b^ ^]^ As shown in Figure 13b, they combined highly polar TiO_2_ and highly conductive TiN to design a twinborn TiO_2_–TiN heterostructure loaded on graphene. Comparing with bare TiO_2_ that has a long diffusion path from the TiO_2_ surface to the nearby carbon substrate, the LiPSs can quickly diffuse to nearby TiN for further conversion after being trapped at the TiO_2_ surface. As a result, the performances of TiO_2_–TiN heterostructures were much better than both pure TiO_2_ and TiN, realizing a high sulfur loading performance of 4.3 mg cm^−2^ and a capacity retention of 67% over 2000 cycles at 1C.

Based on the above concept of combining desired properties to smooth the diffusion of LiPSs, the heterostructure combining metal oxide and metal carbide/nitride/sulfate/phosphide was proposed with the polar character of the former and outstanding catalytic activity of the latter.^[^ ^161^ ^]^ We note that the formed heterojunction can provide a higher adsorption and catalytic activity. For example, in a typical Mn_3_O_4_‐MnP*_x_* heterostructure, the electron redistribution at the interface between Mn_3_O_4_ and MnP*_x_* would lead to the electron accumulation on P atoms. Thus, the Mn can easily accept electrons from LiPSs, displaying a more optimal binding energy and a high catalytic activity.^[^ ^162^ ^]^ Besides sharp interfaces that are present at most heterojunctions, the conjunction could also showcase a transition state. As shown in Figure 13c, the conjunction in the MoO_3_/MoO_2_ heterostructure exhibits a unique transition state of MO*_x_* formed between the MoO_3_ and MoO_2_, which provides a stronger adsorption of LiPSs as compared to MoO_3_ and MoO_2_. This enables Li—S batteries with a high stable cycling performance and a capacity decay of 0.02% per cycle over 850 cycles.^[^ ^103^ ^]^

As stated above, the heterostructure endows the redistribution of electrons, leading to the formation of BIEF with electric positive and negative charged sides, which shows impressively adsorption and redox activity of LiPSs.^[^ ^154^ ^]^ Beyond that, the BIEF can also spatially propel the stepwise conversion. Zhang et al. implemented p‐n heterojunctions by combining n‐type TiO_2_ and p‐type Co_3_O_4_ and found that the BIEF formed at their interface could induce the directional migration of negatively charged polysulfides from p‐type Co_3_O_4_ to n‐type TiO_2_, realizing a spatially optimized distribution of LiPSs (Figure 13d).^[^ ^77^ ^]^ Bridged by the BIEF of p‐n heterojunctions, the interfacial architecture with distinct catalysis could synergistically improve the Li—S performance by spatially enhancing the stepwise conversion of LiPSs. That is, the high catalytic Co_3_O_4_ ensures the precise conversion from S_8_ to Li_2_S_4_, and TiO_2_ can strongly anchor Li_2_S_4_ and further mediate the effective nucleation. As a result, a capacity of 5.5 mAh cm^−2^ could be achieved at the sulfur loading of 5.5 mg cm^−2^ and E/S ratio of 8 µL mg^−1^. Except the effect of BIEF in the heterojunction, the imposed tensile strain in the heterojunction also has great effect to the polysulfide behavior. Chen et al. investigated the exerted internal stress on the MXene (Ti_3_C_2_T*_x_*) after in‐situ spraying of an oxidation layer, and found the formed O—Ti—C in the interface exerts a lattice distortion and enlarge the T—Ti bond of Ti_3_C_2_, resulting in upshift of *d*‐band center of facial Ti atom closer to Fermi level, which strengthened the adsorption and catalytic conversion to LiPSs. After interwoven with CNTs, the hierarchical architecture achieved a high area capacity of 5 mAh cm^−2^ with high sulfur loading of 7 mg cm^−2^ and lean E/S ratio (Figure 13e).^[^ ^160^ ^]^

The heterostructure which combines the advantages of adsorption and catalytic superiority can smooth the LiPSs diffusion in the typical “adsorption‐diffusion‐conversion” process, significantly improving the transfer kinetics in Li—S batteries. Nevertheless, the influence of charge redistribution and other effect in the heteroconjunction is still a matter of investigation. Further development is still needed to elucidate the systematic nature and underlying mechanism of heterojunctions in future research.

## 6 Conclusion and Outlook

To summarize, in this review, we have comprehensively discussed the underlying adsorption and catalytic mechanism of LiPSs in Li—S chemistry. We note that the catalytic property is correlated with the adsorption behavior rather than being independent each other. In this respect, three types of interactions of polysulfide including Li bond, S bond and sulfur‐chain catenation are classified. Especially, we highlight the significance of orbital hybridization which plays an important role in further catalytic analysis. Although the precise mechanism governing the catalytic activity still remains unclear, the Sabatier principle, *d*‐band theory, *d*‐*p* model and Goldilocks principles are introduced here, respectively, which provide valuable guidance for material screening and rational electrocatalysts design.

MOs stand out among metal compounds for their high chemical stability and low cost. There are also other metal compounds that can provide strong and effective affinity to LiPSs, such as metal carbides, sulfides, nitrides and phosphides. However, the above metal compounds usually require strict conditions for synthesis to avoid oxidation, such as high purity of inert atmosphere and high temperature, but still face uncontrollable surface oxidation after being exposed with air.^[^ ^163^ ^]^ It is interesting to note that MOs can also serve as precursor to synthesize above mentioned four metal compounds.^[^ ^12^, ^41^ ^]^ Thus, MOs oxides with high stability and low cost are most promising for applications in Li—S batteries. Moreover, since the exposed surface of MOs is responsible for anchoring, diffusion and catalytic reaction of LiPSs, the nanostructured MOs with controllable exposed surfaces are expected to afford efficient and effective anchoring sites for LiPSs.

Next, different nanostructured MOs based conductive composites applied in Li—S batteries are reviewed. It is found that: 1) most MOs with oxygen termination are able to provide lone pair electrons to form a Li bonds with LiPSs. But some MOs with non‐stoichiometric metal centers present sulfiphilic surfaces, which are inclined to form S bonds; 2) besides chemical composition, different bulk phases and crystal facets can also deliver different adsorption and catalytic activities; and 3) the dual chemical adsorption mode combining both sulfur‐chain catenation and Li/S bond mechanism favors to realize significant adsorption and catalytic properties. In addition, to overcome the sluggish diffusion step in typical “adsorption‐diffusion‐conversion” processes and the low conductivity nature of MOs, constructing OVs and heterostructure are the two main strategies by altering the electronic structure. With many promising accomplishments being achieved, these two strategies showcase potential for the design of high performance Li—S batteries of commercial interest.

Indeed, the change of the electronic structure especially valance state of the catalytic material can regulate the catalytic activity thus making a significant influence on the electrocatalyst's performance. *D*‐band theory provides an explanation that the electron structure influences catalytic activity by shifting the relative position of *d*‐band center. Manufacturing defects, including vacancies, disorders, distortions, boundaries, single atoms, etc., are the common strategies to tune electron structure and further influences the catalytic performance. In the future, controllable tuning of the electronic structures of catalysts, is of crucial importance to realize highly active and efficient catalysts. As such, MOs, with high chemical stability, are most promising and preferable.

Overall, challenges need to be overcome to develop a highly efficient multifunctional catalytic material to realize a high performance that meets the practical requirement. MOs being cost‐efficient, durable, highly polar, environmentally friendly, and possessing tunable properties have the potential to serve as promising catalytic material for the ultimate practical application of high energy density Li—S batteries.

## Conflict of Interest

The authors declare no conflict of interest.

## References

[1] a) W. M. Kang , N. P. Deng , J. G. Ju , Q. X. Li , D. Y. Wu , X. M. Ma , L. Li , M. Naebe , B. W. Cheng , Nanoscale 2016, 8, 16541; 27714087 10.1039/c6nr04923k

[2] a) P. Wang , B. J. Xi , M. Huang , W. H. Chen , J. K. Feng , S. L. Xiong , Adv. Energy Mater. 2021, 11, 2002893;

[3] R. G. Cao , W. Xu , D. P. Lv , J. Xiao , J. G. Zhang , Adv. Energy Mater. 2015, 5, 1402273.

[4] a) X. L. Ji , L. F. Nazar , J. Mater. Chem. 2010, 20, 9821;

[5] a) N. Jayaprakash , J. Shen , S. S. Moganty , A. Corona , L. A. Archer , Angew. Chem., Int. Ed. 2011, 50, 5904; 10.1002/anie.201100637 21591036

[6] a) L. B. Ma , R. P. Chen , G. Y. Zhu , Y. Hu , Y. R. Wang , T. Chen , J. Liu , Z. Jin , ACS Nano 2017, 11, 7274; 28682589 10.1021/acsnano.7b03227

[7] a) N. Kang , Y. X. Lin , L. Yang , D. P. Lu , J. Xiao , Y. Qi , M. Cai , Nat. Commun. 2019, 10, 4597; 31601812 10.1038/s41467-019-12542-6 PMC6787095

[8] S. Dorfler , H. Althues , P. Hartel , T. Abendroth , B. Schumm , S. Kaskel , Joule 2020, 4, 539.

[9] a) R. Wang , J. L. Yang , X. Chen , Y. Zhao , W. G. Zhao , G. Y. Qian , S. N. Li , Y. G. Xiao , H. Chen , Y. S. Ye , G. M. Zhou , F. Pan , Adv. Energy Mater. 2020, 10, 1903550;

[10] a) Z. Z. Du , X. J. Chen , W. Hu , C. H. Chuang , S. Xie , A. J. Hu , W. S. Yan , X. H. Kong , X. J. Wu , H. X. Ji , L. J. Wan , J. Am. Chem. Soc. 2019, 141, 3977; 30764605 10.1021/jacs.8b12973

[11] a) X. Luo , X. B. Lu , X. D. Chen , Y. Chen , C. Y. Yu , D. W. Su , G. X. Wang , L. F. Cui , J. Energy Chem. 2020, 50, 63;

[12] J. B. Zhou , X. J. Liu , L. Q. Zhu , J. Zhou , Y. Guan , L. Chen , S. W. Niu , J. Y. Cai , D. Sun , Y. C. Zhu , J. Du , G. M. Wang , Y. T. Qian , Joule 2018, 2, 2681.

[13] S. Feng , Z. H. Fu , X. Chen , Q. Zhang , Infomat 2022, 4, e12304.

[14] X. Liu , J. Q. Huang , Q. Zhang , L. Q. Mai , Adv. Mater. 2017, 29, 1601759. 10.1002/adma.201601759 28160327

[15] a) H. J. Peng , G. Zhang , X. Chen , Z. W. Zhang , W. T. Xu , J. Q. Huang , Q. Zhang , Angew. Chem., Int. Ed. 2016, 55, 12990; 10.1002/anie.201605676 27513988

[16] Y. K. Wang , R. F. Zhang , J. Chen , H. Wu , S. Y. Lu , K. Wang , H. L. Li , C. J. Harris , K. Xi , R. V. Kumar , S. J. Ding , Adv. Energy Mater. 2019, 9, 1900953.

[17] a) Y. K. Wang , R. F. Zhang , Z. H. Sun , H. Wu , S. Y. Lu , J. N. Wang , W. Yu , J. M. Liu , G. X. Gao , S. J. Ding , Adv. Mater. Interfaces 2020, 7, 1902092;

[18] a) Z. Zhang , D. Luo , G. R. Li , R. Gao , M. Li , S. Li , L. Zhao , H. Z. Dou , G. B. Wen , S. Sy , Y. F. Hu , J. D. Li , A. P. Yu , Z. W. Chen , Matter 2020, 3, 920;

[19] H. L. Pan , X. L. Wei , W. A. Henderson , Y. Y. Shao , J. Z. Chen , P. Bhattacharya , J. Xiao , J. Liu , Adv. Energy Mater. 2015, 5, 1500113.

[20] L. C. Yin , J. Liang , G. M. Zhou , F. Li , R. Saito , H. M. Cheng , Nano Energy 2016, 25, 203.

[21] a) Q. F. Zhang , Y. P. Wang , Z. W. Seh , Z. H. Fu , R. F. Zhang , Y. Cui , Nano Lett. 2015, 15, 3780; 25961805 10.1021/acs.nanolett.5b00367

[22] a) Z. W. Seh , Q. F. Zhang , W. Y. Li , G. Y. Zheng , H. B. Yao , Y. Cui , Chem. Sci. 2013, 4, 3673;

[23] H. Margenau , Rev. Mod. Phys. 1939, 11, 1.

[24] a) J. X. Song , M. L. Gordin , T. Xu , S. R. Chen , Z. X. Yu , H. Sohn , J. Lu , Y. Ren , Y. H. Duan , D. H. Wang , Angew. Chem., Int. Ed. 2015, 54, 4325; 10.1002/anie.201411109 25663183

[25] D. N. Shigorin , Spectrochim. Acta 1959, 14, 198.

[26] A. B. Sannigrahi , T. Kar , B. G. Niyogi , P. Hobza , P. v. R. Schleyer , Chem. Rev. 1990, 90, 1061.

[27] R. G. Pearson , J. Chem. Educ. 1968, 45, 643.

[28] X. Chen , H. J. Peng , R. Zhang , T. Z. Hou , J. Q. Huang , B. Li , Q. Zhang , ACS Energy Lett. 2017, 2, 795.

[29] Q. Pang , X. Liang , C. Y. Kwok , L. F. Nazar , J. Electrochem. Soc. 2015, 162, A2567.

[30] Z. Y. Han , S. Y. Zhao , J. W. Xiao , X. W. Zhong , J. Z. Sheng , W. Lv , Q. F. Zhang , G. M. Zhou , H. M. Cheng , Adv. Mater. 2021, 33, 2105947. 10.1002/adma.202105947 34569660

[31] S. S. Zhang , Electrochim. Acta 2012, 70, 344.

[32] L. L. Peng , Z. Y. Wei , C. Z. Wan , J. Li , Z. Chen , D. Zhu , D. Baumann , H. T. Liu , C. S. Allen , X. Xu , A. I. Kirkland , I. Shakir , Z. Almutairi , S. Tolbert , B. Dunn , Y. Huang , P. Sautet , X. F. Duan , Nat. Catal. 2020, 3, 762.

[33] a) Q. Pang , C. Y. Kwok , D. Kundu , X. Liang , L. F. Nazar , Joule 2019, 3, 136;

[34] a) Y. W. Wang , W. J. Qiu , E. H. Song , F. Gu , Z. H. Zheng , X. L. Zhao , Y. Q. Zhao , J. J. Liu , W. Q. Zhang , Natl. Sci. Rev. 2018, 5, 327;

[35] a) T. G. Jeong , D. S. Choi , H. Song , J. Choi , S. A. Park , S. H. Oh , H. Kim , Y. Jung , Y. T. Kim , ACS Energy Lett. 2017, 2, 327;

[36] a) A. J. Medford , A. Vojvodic , J. S. Hummelshoj , J. Voss , F. Abild‐Pedersen , F. Studt , T. Bligaard , A. Nilsson , J. K. Norskov , J. Catal. 2015, 328, 36;

[37] a) B. Hammer , J. K. Norskov , Nature 1995, 376, 238;

[38] a) Z. H. Shen , Z. L. Zhang , M. Li , Y. F. Yuan , Y. Zhao , S. Zhang , C. L. Zhong , J. Zhu , J. Lu , H. G. Zhang , ACS Nano 2020, 14, 6673; 32463691 10.1021/acsnano.9b09371

[39] P. Zeng , C. Liu , C. Cheng , C. Yuan , K. H. Dai , J. Mao , L. R. Zheng , J. Zhang , L. Y. Chang , S. C. Haw , T. S. Chan , H. P. Lin , L. Zhang , J. Mater. Chem. A 2021, 9, 18526.

[40] Z. X. Shi , M. Li , J. Y. Sun , Z. W. Chen , Adv. Energy Mater. 2021, 11, 2100332.

[41] J. D. Shen , X. J. Xu , J. Liu , Z. B. Liu , F. K. Li , R. Z. Hu , J. W. Liu , X. H. Hou , Y. Z. Feng , Y. Yu , M. Zhu , ACS Nano 2019, 13, 8986. 31356051 10.1021/acsnano.9b02903

[42] a) Z. X. Zhao , Z. L. Yi , H. J. Li , R. Pathak , Z. W. Yang , X. M. Wang , Q. Q. Qiao , Nano Energy 2021, 81, 105621;

[43] Q. Cheng , W. H. Xu , S. Y. Qin , S. Das , T. W. Jin , A. J. Li , A. C. Li , B. Y. Qie , P. C. Yao , H. W. Zhai , C. M. Shi , X. Yong , Y. Yang , Angew. Chem., Int. Ed. 2019, 58, 5557. 10.1002/anie.201812611 30779275

[44] H. L. Ye , J. G. Sun , S. L. Zhang , T. R. Zhang , Y. Zhao , C. Y. Song , Q. F. Yao , J. Y. Lee , Chem. Eng. J. 2021, 410, 128284.

[45] a) M. Zhao , H. J. Peng , J. Y. Wei , J. Q. Huang , B. Q. Li , H. Yuan , Q. Zhang , Small Methods 2020, 4, 1900344;

[46] F. Y. Fan , W. C. Carter , Y. M. Chiang , Adv. Mater. 2015, 27, 5203. 26257297 10.1002/adma.201501559

[47] Z. Lin , Z. C. Liu , W. J. Fu , N. J. Dudney , C. D. Liang , Angew. Chem., Int. Ed. 2013, 52, 7460. 10.1002/anie.201300680 23737078

[48] X. Liang , C. Hart , Q. Pang , A. Garsuch , T. Weiss , L. F. Nazar , Nat. Commun. 2015, 6, 5682. 25562485 10.1038/ncomms6682

[49] H. Stamm , H. Wintzer , Ber. Dtsch. Chem. Ges. 1938, 71, 2212.

[50] X. Liang , Y. Rangom , C. Y. Kwok , Q. Pang , L. F. Nazar , Adv. Mater. 2017, 29, 1603040. 10.1002/adma.201603040 27859697

[51] a) X. Liang , C. Y. Kwok , F. Lodi‐Marzano , Q. Pang , M. Cuisinier , H. Huang , C. J. Hart , D. Houtarde , K. Kaup , H. Sommer , T. Brezesinski , J. Janek , L. F. Nazar , Adv. Energy Mater. 2016, 6, 1501636;

[52] H. Tang , W. L. Li , L. M. Pan , K. J. Tu , F. Du , T. Qiu , J. Yang , C. P. Cullen , N. McEvoy , C. F. Zhang , Adv. Funct. Mater. 2019, 29, 1901907.

[53] Y. Chen , T. Y. Wang , H. J. Tian , D. W. Su , Q. Zhang , G. X. Wang , Adv. Mater. 2021, 33, 2003666. 10.1002/adma.202003666 34096100

[54] J. C. Vedrine , Catalysts 2017, 7, 341.

[55] Y. J. Choi , B. S. Jung , D. J. Lee , J. H. Jeong , K. W. Kim , H. J. Ahn , K. K. Cho , H. B. Gu , Phys. Scr. 2007, T129, 62.

[56] X. L. Ji , S. Evers , R. Black , L. F. Nazar , Nat. Commun. 2011, 2, 325. 21610728 10.1038/ncomms1293

[57] M. S. Song , S. C. Han , H. S. Kim , J. H. Kim , K. T. Kim , Y. M. Kang , H. J. Ahn , S. X. Dou , J. Y. Lee , J. Electrochem. Soc. 2004, 151, A791.

[58] S. Evers , T. Yim , L. F. Nazar , J. Phys. Chem. C 2012, 116, 19653.

[59] a) X. Song , T. Gao , S. Q. Wang , Y. Bao , G. P. Chen , L. X. Ding , H. H. Wang , J. Power Sources 2017, 356, 172;

[60] N. Ding , L. Zhou , C. W. Zhou , D. S. Geng , J. Yang , S. W. Chien , Z. L. Liu , M. F. Ng , A. S. Yu , T. S. A. Hor , M. B. Sullivan , Y. Zong , Sci. Rep. 2016, 6, 33154. 27629986 10.1038/srep33154 PMC5024100

[61] B. Ding , G. Y. Xu , L. F. Shen , P. Nie , P. F. Hu , H. Dou , X. G. Zhang , J. Mater. Chem. A 2013, 1, 14280.

[62] Z. Zhang , Q. Li , K. Zhang , W. Chen , Y. Q. Lai , J. Li , J. Power Sources 2015, 290, 159.

[63] Z. B. Xiao , Z. Yang , L. Wang , H. G. Nie , M. E. Zhong , Q. Q. Lai , X. J. Xu , L. J. Zhang , S. M. Huang , Adv. Mater. 2015, 27, 2891. 25820906 10.1002/adma.201405637

[64] Z. Zhang , Q. Li , S. F. Jiang , K. Zhang , Y. Q. Lai , J. Li , Chem. ‐ Eur. J. 2015, 21, 1343. 25413990 10.1002/chem.201404686

[65] W. J. Xue , Q. B. Yan , G. Y. Xu , L. M. Suo , Y. M. Chen , C. Wang , C. A. Wang , J. Li , Nano Energy 2017, 38, 12.

[66] L. Q. Yang , G. C. Li , X. Jiang , T. R. Zhang , H. B. Lin , J. Y. Lee , J. Mater. Chem. A 2017, 5, 12506.

[67] M. M. Fang , Z. M. Chen , Y. Liu , J. P. Quan , C. Yang , L. C. Zhu , Q. B. Xu , Q. Xu , J. Mater. Chem. A 2018, 6, 1630.

[68] W. L. Wu , J. Pu , J. Wang , Z. H. Shen , H. Y. Tang , Z. T. Deng , X. Y. Tao , F. Pan , H. G. Zhang , Adv. Energy Mater. 2018, 8, 1702373.

[69] H. B. Ding , Q. F. Zhang , Z. M. Liu , J. Wang , R. F. Ma , L. Fan , T. Wang , J. G. Zhao , J. M. Ge , X. L. Lu , X. Z. Yu , B. G. Lu , Electrochim. Acta 2018, 284, 314.

[70] R. Q. Liu , Z. W. Liu , W. H. Liu , Y. J. Liu , X. J. Lin , Y. Li , P. Li , Z. D. Huang , X. M. Feng , L. S. Yu , D. Wang , Y. W. Ma , W. Huang , Small 2019, 15, 1804533.

[71] L. Jiao , C. Zhang , C. N. Geng , S. C. Wu , H. Li , W. Lv , Y. Tao , Z. J. Chen , G. M. Zhou , J. Li , G. W. Ling , Y. Wan , Q. H. Yang , Adv. Energy Mater. 2019, 9, 1900219.

[72] G. L. Chen , W. T. Zhong , Y. S. Li , Q. Deng , X. Ou , Q. C. Pan , X. W. Wang , X. H. Xiong , C. H. Yang , M. L. Liu , ACS Appl. Mater. Interfaces 2019, 11, 5055. 30656928 10.1021/acsami.8b19501

[73] X. Q. Zhang , W. Yuan , Y. Yang , Y. Chen , Z. H. Tang , C. Wang , Y. H. Yuan , Y. T. Ye , Y. P. Wu , Y. Tang , Small 2020, 16, 2005998. 10.1002/smll.202005998 33258313

[74] S. Y. Zhou , J. Y. Hu , S. G. Liu , J. X. Lin , J. Cheng , T. Mei , X. B. Wang , H. G. Liao , L. Huang , S. G. Sun , Nano Energy 2020, 72, 104680.

[75] X. L. Zhang , P. Zhang , S. J. Zhang , Y. S. Zhang , R. H. Hou , K. L. Liu , F. J. Miao , G. S. Shao , J. Energy Chem. 2020, 51, 378.

[76] N. R. Li , L. H. Yu , J. Y. Xi , Small 2021, 17, 2103001.

[77] H. T. Li , C. Chen , Y. Y. Yan , T. R. Yan , C. Cheng , D. Sun , L. Zhang , Adv. Mater. 2021, 33, 2105067. 10.1002/adma.202105067 34632643

[78] Y. Q. Feng , H. Liu , Y. Liu , F. W. Zhao , J. Q. Li , X. M. He , J. Energy Chem. 2021, 62, 508.

[79] Z. Li , J. T. Zhang , X. W. Lou , Angew. Chem., Int. Ed. 2015, 54, 12886. 10.1002/anie.201506972 26349817

[80] M. Yan , Y. Zhang , Y. Li , Y. Q. Huo , Y. Yu , C. Wang , J. Jin , L. H. Chen , T. Hasan , B. J. Wang , B. L. Su , J. Mater. Chem. A 2016, 4, 9403.

[81] Y. Li , D. X. Ye , W. Liu , B. Shi , R. Guo , H. B. Zhao , H. J. Pei , J. Q. Xu , J. Y. Xie , ACS Appl. Mater. Interfaces 2016, 8, 28566. 27472481 10.1021/acsami.6b04270

[82] Y. Q. Lai , P. Wang , F. R. Qin , M. Xu , J. Li , K. Zhang , Z. Zhang , Energy Storage Mater. 2017, 9, 179.

[83] Q. Liu , J. H. Zhang , S. A. He , R. J. Zou , C. T. Xu , Z. Cui , X. J. Huang , G. Q. Guan , W. L. Zhang , K. B. Xu , J. Q. Hu , Small 2018, 14, 1703816. 10.1002/smll.201703816 29665267

[84] M. F. Chen , Q. Lu , S. X. Jiang , C. Huang , X. Y. Wang , B. Wu , K. X. Xiang , Y. T. Wu , Chem. Eng. J. 2018, 335, 831.

[85] N. Wang , S. K. Peng , X. Chen , J. X. Wang , C. Wang , X. Qi , S. L. Dai , S. J. Yan , RSC Adv. 2019, 9, 6346. 35517254 10.1039/c9ra00292h PMC9060960

[86] H. Zhang , Q. Qi , P. G. Zhang , W. Zheng , J. Chen , A. G. Zhou , W. B. Tian , W. Zhang , Z. M. Sun , ACS Appl. Energy Mater. 2019, 2, 705.

[87] H. Chen , W. D. Dong , F. J. Xia , Y. J. Zhang , M. Yan , J. P. Song , W. Zou , Y. Liu , Z. Y. Hu , J. Liu , Y. Li , H. E. Wang , L. H. Chen , B. L. Su , Chem. Eng. J. 2020, 381, 122746.

[88] Y. J. Zhang , X. L. Liu , L. Wu , W. D. Dong , F. J. Xia , L. D. Chen , N. Zhou , L. X. Xia , Z. Y. Hu , J. Liu , H. S. H. Mohamed , Y. Li , Y. Zhao , L. H. Chen , B. L. Su , J. Mater. Chem. A 2020, 8, 2741.

[89] H. Pan , Z. B. Cheng , X. Zhang , K. Wan , J. Fransaer , J. S. Luo , M. Wubbenhorst , J. Mater. Chem. A 2020, 8, 21824.

[90] Z. H. Chen , Y. X. Hu , W. Liu , F. Yu , X. F. Yu , T. Mei , L. Yu , X. B. Wang , ACS Appl. Mater. Interfaces 2021, 13, 38394. 34370432 10.1021/acsami.1c10958

[91] J. R. He , L. Luo , Y. F. Chen , A. Manthiram , Adv. Mater. 2017, 29, 1702707. 10.1002/adma.201702707 28692775

[92] Y. M. Liu , X. Y. Qin , S. Q. Zhang , G. M. Liang , F. Y. Kang , G. H. Chen , B. H. Li , ACS Appl. Mater. Interfaces 2018, 10, 26264. 30004216 10.1021/acsami.8b07316

[93] K. Lu , H. Zhang , S. Y. Gao , H. Y. Ma , J. Z. Chen , Y. W. Cheng , Adv. Funct. Mater. 2019, 29, 1807309.

[94] Z. Su , M. Q. Chen , Y. K. Pan , Y. J. Liu , H. Xu , Y. Y. Zhang , D. H. Long , J. Mater. Chem. A 2020, 8, 24117.

[95] S. S. Xin , J. Li , H. T. Cui , Y. Y. Liu , H. Y. Wei , Y. Y. Zhong , M. R. Wang , Chem. Eng. J. 2021, 410, 128153.

[96] Y. G. Zhang , W. L. Qiu , Y. Zhao , Y. Wang , Z. Bakenov , X. Wang , Chem. Eng. J. 2019, 375, 122055.

[97] N. X. Shi , B. J. Xi , Z. Y. Feng , F. F. Wu , D. H. Wei , J. Liu , S. L. Xiong , J. Mater. Chem. A 2019, 7, 4009.

[98] Z. X. Jian , S. C. Zhang , X. G. Guan , J. J. Li , H. L. Li , W. X. Wang , Y. L. Xing , H. Z. Xu , RSC Adv. 2020, 10, 32966. 35516468 10.1039/d0ra04986g PMC9056673

[99] J. Xu , W. X. Zhang , Y. Chen , H. B. Fan , D. W. Su , G. X. Wang , J. Mater. Chem. A 2018, 6, 2797.

[100] R. P. Liu , F. Guo , X. F. Zhang , J. L. Yang , M. Y. Li , M. M. Wu , L. Hang , F. Ming , Z. Lei , ACS Appl. Energy Mater. 2019, 2, 1348.

[101] S. Jiao , T. Y. Ding , R. Zhai , Y. P. Wu , S. Chen , W. Wei , Nanoscale 2020, 12, 13377. 32347276 10.1039/d0nr01239d

[102] L. Zhou , H. Li , X. C. Wu , Y. Zhang , D. L. Danilov , R. A. Eichel , P. H. L. Notten , ACS Appl. Energy Mater. 2019, 2, 8153.

[103] W. W. Yang , Y. Wei , Q. Chen , S. J. Qin , J. H. Zuo , S. D. Tan , P. B. Zhai , S. Q. Cui , H. W. Wang , C. Q. Jin , J. Xiao , W. Liu , J. X. Shang , Y. J. Gong , J. Mater. Chem. A 2020, 8, 15816.

[104] C. Choi , D. Y. Lee , J. B. Park , D. W. Kim , ACS Sustainable Chem. Eng. 2020, 8, 15134.

[105] H. X. Wang , B. Zhang , X. Q. Zeng , L. J. Yan , J. C. Zheng , M. Ling , Y. Hou , Y. Y. Lu , C. D. Liang , J. Power Sources 2020, 473, 228588.

[106] J. Y. Liu , M. F. Zhu , Z. H. Shen , T. L. Han , T. Si , C. Q. Hu , H. G. Zhang , Small 2021, 17, 2103051. 10.1002/smll.202103051 34510738

[107] W. L. Wei , J. M. Li , Q. Wang , D. Liu , J. Y. Niu , P. Liu , ACS Appl. Mater. Interfaces 2020, 12, 6362. 31913593 10.1021/acsami.9b18426

[108] X. W. Liu , Z. H. Li , X. B. Liao , X. F. Hong , Y. Li , C. Zhou , Y. Zhao , X. Xu , L. Q. Mai , J. Mater. Chem. A 2020, 8, 12106.

[109] Y. Guo , Y. Zhang , Y. Zhang , M. W. Xiang , H. Wu , H. K. Liu , S. X. Dou , J. Mater. Chem. A 2018, 6, 19358.

[110] F. Wang , X. Ding , R. Y. Shi , M. R. Li , Y. M. Lei , Z. B. Lei , G. S. Jiang , F. Xu , H. Q. Wang , L. C. Jia , R. B. Jiang , Z. H. Liu , J. Sun , J. Mater. Chem. A 2019, 7, 10494.

[111] M. W. Xiang , H. Wu , H. Liu , J. Huang , Y. F. Zheng , L. Yang , P. Jing , Y. Zhang , S. X. Dou , H. K. Liu , Adv. Funct. Mater. 2017, 27, 1702573.

[112] X. B. Jia , B. S. Liu , J. H. Liu , S. H. Zhang , Z. J. Sun , X. He , H. D. Li , G. F. Wang , H. X. Chang , RSC Adv. 2021, 11, 10753. 35423542 10.1039/d1ra00216c PMC8695830

[113] Y. Q. Tao , Y. J. Wei , Y. Liu , J. T. Wang , W. M. Qiao , L. C. Ling , D. H. Long , Energy Environ. Sci. 2016, 9, 3230.

[114] K. Z. Lv , P. F. Wang , C. Wang , Z. H. Shen , Z. D. Lu , H. G. Zhang , M. B. Zheng , P. He , H. S. Zhou , Small 2020, 16, 2000870.

[115] F. G. Sun , J. T. Wang , D. H. Long , W. M. Qiao , L. C. Ling , C. X. Lv , R. Cai , J. Mater. Chem. A 2013, 1, 13283.

[116] Y. Z. Song , W. Zhao , N. Wei , L. Zhang , F. Ding , Z. F. Liu , J. Y. Sun , Nano Energy 2018, 53, 432.

[117] WHO , inIARC‐Evaluation of Carcinogenic Risks to Humans, International Agency for Research on Cancer, Lyon Cedex, France, 2010, 193–276.

[118] M. P. Yu , J. S. Ma , H. Q. Song , A. J. Wang , F. Y. Tian , Y. S. Wang , H. Qiu , R. M. Wang , Energy Environ. Sci. 2016, 9, 1495.

[119] M. M. Zhen , K. L. Jiang , S. Q. Guo , B. X. Shen , H. L. Liu , Nano Res. 2022, 15, 933.

[120] X. Zhong , I. Rungger , P. Zapol , O. Heinonen , Phys. Rev. B 2015, 91, 115143.

[121] Q. Pang , D. Kundu , M. Cuisinier , L. F. Nazar , Nat. Commun. 2014, 5, 4759. 25154399 10.1038/ncomms5759

[122] X. Y. Tao , J. G. Wang , Z. G. Ying , Q. X. Cai , G. Y. Zheng , Y. P. Gan , H. Huang , Y. Xia , C. Liang , W. K. Zhang , Y. Cui , Nano Lett. 2014, 14, 5288. 25089648 10.1021/nl502331f

[123] a) M. T. Liu , S. Jhulki , Z. F. Sun , A. Magasinski , C. Hendrix , G. Yushin , Nano Energy 2021, 79, 105428;

[124] C. A. Zhou , X. Y. Sun , W. Yan , Y. Z. Zuo , J. J. Zhang , Chem. ‐ Asian J. 2022, 17, e202200328. 35586952 10.1002/asia.202200328

[125] Y. Liu , J. Wei , Y. X. Tian , S. Q. Yan , J. Mater. Chem. A 2015, 3, 19000.

[126] L. B. Ni , Z. Wu , G. J. Zhao , C. Y. Sun , C. Q. Zhou , X. X. Gong , G. W. Diao , Small 2017, 13, 1603466. 10.1002/smll.201603466 28134468

[127] a) W. Dong , L. Q. Meng , X. D. Hong , S. Z. Liu , D. Shen , Y. K. Xia , S. B. Yang , Molecules 2020, 25, 1989; 32340399 10.3390/molecules25081989 PMC7221920

[128] a) S. G. Deng , Q. H. Li , Y. H. Chen , C. Wang , H. B. Zhao , J. Q. Xu , J. H. Wu , X. Y. Yao , Inorg. Chem. Front. 2021, 8, 1771;

[129] G. X. Liu , K. Feng , H. T. Cui , J. Li , Y. Y. Liu , M. R. Wang , Chem. Eng. J. 2020, 381, 122652.

[130] a) X. R. He , Y. J. Zhang , L. F. Yang , J. L. Zhao , H. T. Li , Y. B. Gao , B. Wang , X. D. Guo , Acta Metall. Sin. (Engl. Lett.) 2021, 34, 410;

[131] J. S. Yeon , Y. H. Ko , T. H. Park , H. Park , J. Kim , H. S. Park , Energy Environ. Mater. 2022, 5, 555.

[132] a) S. J. M. Rosid , S. Toemen , M. M. A. Iqbal , W. A. W. Abu Bakar , W. N. A. W. Mokhtar , M. M. A. Aziz , Environ. Sci. Pollut. Res. 2019, 26, 36124; 10.1007/s11356-019-06607-8 31748998

[133] B. Jiang , Y. Qiu , D. Tian , Y. Zhang , X. Q. Song , C. H. Zhao , M. X. Wang , X. Sun , H. H. Huang , C. Y. Zhao , H. Zhou , A. S. Chen , L. S. Fan , N. Q. Zhang , Adv. Energy Mater. 2021, 11, 2102995.

[134] H. Cheng , S. C. Zhang , S. Y. Li , C. Gao , S. H. Zhao , Y. Y. Lu , M. Wang , Small 2022, 18, 2202557. 10.1002/smll.202202557 35718880

[135] Y. Chen , J. Y. Li , X. B. Kong , Y. Y. Zhang , Y. J. Zhang , J. B. Zhao , ACS Sustainable Chem. Eng. 2021, 9, 10392.

[136] J. Y. Wang , G. R. Li , D. Luo , Y. G. Zhang , Y. Zhao , G. F. Zhou , L. L. Shui , X. Wang , Z. W. Chen , Adv. Energy Mater. 2020, 10, 2002076.

[137] M. H. Li , S. Ji , X. G. Ma , H. Wang , X. Y. Wang , V. Linkov , R. F. Wang , ACS Appl. Mater. Interfaces 2022, 14, 16310. 35348314 10.1021/acsami.2c02558

[138] a) G. Pacchioni , ChemPhysChem 2003, 4, 1041; 14595999 10.1002/cphc.200300835

[139] X. Y. Pan , M. Q. Yang , X. Z. Fu , N. Zhang , Y. J. Xu , Nanoscale 2013, 5, 3601. 23532413 10.1039/c3nr00476g

[140] J. W. Wan , W. X. Chen , C. Y. Jia , L. R. Zheng , J. C. Dong , X. S. Zheng , Y. Wang , W. S. Yan , C. Chen , Q. Peng , D. S. Wang , Y. D. Li , Adv. Mater. 2018, 30, 1705369.

[141] a) J. Wang , D. N. Tafen , J. P. Lewis , Z. L. Hong , A. Manivannan , M. J. Zhi , M. Li , N. Q. Wu , J. Am. Chem. Soc. 2009, 131, 12290; 19705915 10.1021/ja903781h

[142] L. Sharma , P. Kumar , A. Halder , ChemElectroChem 2019, 6, 3420.

[143] H. C. Wang , C. Y. Fan , Y. P. Zheng , X. H. Zhang , W. H. Li , S. Y. Liu , H. Z. Sun , J. P. Zhang , L. N. Sun , X. L. Wu , Chem. ‐ Eur. J. 2017, 23, 9666. 28508401 10.1002/chem.201701580

[144] G. M. Wang , Y. Yang , Y. C. Ling , H. Y. Wang , X. H. Lu , Y. C. Pu , J. Z. Zhang , Y. X. Tong , Y. Li , J. Mater. Chem. A 2016, 4, 2849.

[145] G. C. Xi , S. X. Ouyang , P. Li , J. H. Ye , Q. Ma , N. Su , H. Bai , C. Wang , Angew. Chem., Int. Ed. 2012, 51, 2395. 10.1002/anie.201107681 22282345

[146] C. Y. Fan , C. Chen , J. Wang , X. X. Fu , Z. M. Ren , G. D. Qian , Z. Y. Wang , Sci. Rep. 2015, 5, 11712. 26133789 10.1038/srep11712 PMC4488957

[147] Y. Y. Zhu , Q. Ling , Y. F. Liu , H. Wang , Y. F. Zhu , Appl. Catal., B 2016, 187, 204.

[148] a) Y. X. Zhao , Y. F. Zhao , R. Shi , B. Wang , G. I. N. Waterhouse , L. Z. Wu , C. H. Tung , T. R. Zhang , Adv. Mater. 2019, 31, 1806482; 10.1002/adma.201806482 30828882

[149] H. B. Lin , S. L. Zhang , T. R. Zhang , H. L. Ye , Q. F. Yao , G. W. Zheng , J. Y. Lee , Adv. Energy Mater. 2018, 8, 1801868.

[150] H. E. Wang , K. L. Yin , N. Qin , X. Zhao , F. J. Xia , Z. Y. Hu , G. L. Guo , G. Z. Cao , W. J. Zhang , J. Mater. Chem. A 2019, 7, 10346.

[151] J. W. Wu , Q. Y. Ma , C. Lian , Y. Yuan , D. H. Long , Chem. Eng. J. 2019, 370, 556.

[152] L. L. Xu , H. Y. Zhao , M. Z. Sun , B. L. Huang , J. W. Wang , J. L. Xia , N. Li , D. D. Yin , M. Luo , F. Luo , Y. P. Du , C. H. Yan , Angew. Chem., Int. Ed. 2019, 58, 11491. 10.1002/anie.201905852 31206953

[153] K. He , T. T. Tsega , X. Liu , J. T. Zai , X. H. Li , X. J. Liu , W. H. Li , N. Ali , X. F. Qian , Angew. Chem., Int. Ed. 2019, 58, 11903. 10.1002/anie.201905281 31209961

[154] Y. J. Li , W. Y. Wang , B. Zhang , L. Fu , M. T. Wan , G. C. Li , Z. Cai , S. B. Tu , X. R. Duan , Z. W. Seh , J. J. Jiang , Y. M. Sun , Nano Lett. 2021, 21, 6656. 34291943 10.1021/acs.nanolett.1c02161

[155] B. Zhang , C. Luo , Y. Q. Deng , Z. J. Huang , G. M. Zhou , W. Lv , Y. B. He , Y. Wan , F. Y. Kang , Q. H. Yang , Adv. Energy Mater. 2020, 10, 2000091.

[156] Y. P. Gao , L. B. Wang , A. G. Zhou , Z. Y. Li , J. K. Chen , H. Bala , Q. K. Hu , X. X. Cao , Mater. Lett. 2015, 150, 62.

[157] a) C. F. Zhang , S. J. Kim , M. Ghidiu , M. Q. Zhao , M. W. Barsoum , V. Nicolosi , Y. Gogotsi , Adv. Funct. Mater. 2016, 26, 4143;

[158] a) Y. P. Lv , S. B. Duan , R. M. Wang , Prog. Nat. Sci.: Mater. Int. 2020, 30, 1;

[159] D. Q. Cai , J. L. Yang , T. Liu , S. X. Zhao , G. Z. Cao , Nano Energy 2021, 89, 106452.

[160] X. Wang , D. Luo , J. Y. Wang , Z. H. Sun , G. L. Cui , Y. X. Chen , T. Wang , L. R. Zheng , Y. Zhao , L. L. Shui , G. F. Zhou , K. Kempa , Y. G. Zhang , Z. W. Chen , Angew. Chem., Int. Ed. 2021, 60, 2371. 10.1002/anie.202011493 33398902

[161] a) Y. Z. Song , W. Zhao , L. Kong , L. Zhang , X. Y. Zhu , Y. L. Shao , F. Ding , Q. Zhang , J. Y. Sun , Z. F. Liu , Energy Environ. Sci. 2018, 11, 2620;

[162] K. Guo , G. Qu , J. Li , H. C. Xia , W. F. Yan , J. W. Fu , P. F. Yuan , J. A. Zhang , Energy Storage Mater. 2021, 36, 496.

[163] a) K. Xi , D. Q. He , C. Harris , Y. K. Wang , C. Lai , H. L. Li , P. R. Coxon , S. J. Ding , C. Wang , R. V. Kumar , Adv. Sci. 2019, 6, 1800815; 10.1002/advs.201800815 PMC6425436 30937253

